# MBR-SIFT: A mirror reflected invariant feature descriptor using a binary representation for image matching

**DOI:** 10.1371/journal.pone.0178090

**Published:** 2017-05-18

**Authors:** Mingzhe Su, Yan Ma, Xiangfen Zhang, Yan Wang, Yuping Zhang

**Affiliations:** 1 College of Information, Mechanical and Electrical Engineering, Shanghai Normal University, Shanghai, China; 2 Mathématiques, Informatique, Télécommunications de Toulouse, Université Paul Sabatier, Toulouse, France; National University of Defense Technology College of Mechatronic Engineering and Automation, CHINA

## Abstract

The traditional scale invariant feature transform (SIFT) method can extract distinctive features for image matching. However, it is extremely time-consuming in SIFT matching because of the use of the Euclidean distance measure. Recently, many binary SIFT (BSIFT) methods have been developed to improve matching efficiency; however, none of them is invariant to mirror reflection. To address these problems, in this paper, we present a horizontal or vertical mirror reflection invariant binary descriptor named MBR-SIFT, in addition to a novel image matching approach. First, 16 cells in the local region around the SIFT keypoint are reorganized, and then the 128-dimensional vector of the SIFT descriptor is transformed into a reconstructed vector according to eight directions. Finally, the MBR-SIFT descriptor is obtained after binarization and reverse coding. To improve the matching speed and accuracy, a fast matching algorithm that includes a coarse-to-fine two-step matching strategy in addition to two similarity measures for the MBR-SIFT descriptor are proposed. Experimental results on the UKBench dataset show that the proposed method not only solves the problem of mirror reflection, but also ensures desirable matching accuracy and speed.

## Introduction

The local feature point has been successfully used in pattern recognition and computer vision applications, such as image retrieval [1], object recognition [2], gesture recognition [3], texture recognition [4], 3-D reconstruction [5], building panoramas [6], and wide baseline matching [7,8]. Image matching based on local features generally consists of three stages: feature point extraction, description, and matching. In feature point extraction, reliable points of interest in the image are extracted as feature points. A good descriptor should be robust to photometric transformations, such as brightness and highlight, while being invariant to geometrical transformations, such as rotation, scaling, viewpoint, and reflection [9].

Until recently, numerous feature descriptors have been proposed, of which the scale invariant feature transform (SIFT) descriptor proposed by Lowe [10] is one of the most successful and popular local image feature descriptors. The SIFT descriptor, which is generated with the gradient distribution of the local region, was proven to be the best local invariant feature descriptor by Mikolajczyk and Schmid [11]. However, its matching inefficiency slows down the entire process. Much research has been conducted on improving the SIFT algorithm. The PCA-SIFT [12] descriptor improves the efficiency of the SIFT algorithm by reducing the dimension of the SIFT descriptor vector from 128 to 36. Additionally, GLOH [11] is an extension of the SIFT descriptor that is designed to increase its robustness and distinctiveness, to a certain extent. Morel and Yu [13] proposed an affine SIFT, which simulates all the distortions caused by variations in the direction of a camera’s optical axis.

In the matching procedure, the 128-dimensional (128-D) descriptors of all keypoints in two images are extracted. The 128-D descriptor of each keypoint in the first image is compared with that of the second image. The Euclidean distance is used as the similarity measurement of the two descriptors to locate the nearest matching keypoint. However, the SIFT algorithm usually generates hundreds to thousands of keypoints for each image. Hence, the SIFT features could be numerous in a large-scale image database. Moreover, the distance computation involves calculating the square root. Thus, image matching in the SIFT method for a large-scale image database would be extremely time-consuming. To solve this problem, several binary SIFT (BSIFT) methods, which convert the SIFT descriptors to a binary representation, have been proposed in the last few years. The Hamming distance is used to measure the distance between two BSIFT descriptors, which takes advantage of bit-wise operations instead of the root mean square, and leads to a significant decrease in feature matching time. Ni [14] first proposed a binary string approach for SIFT keypoints. His method exploited the Hamming distance to measure the similarity of two BSIFT vectors. Chen et al. [15] proposed comparing the absolute difference between two adjacent values in a descriptor with the threshold, and then representing the comparison result with binary digits (zero or one), which generated a 128-bit BSIFT descriptor string. This approach was simple, while drastically decreasing the matching time; however, the matching accuracy rate also decreased. Zhou et al. [16] compared the 128 values of the SIFT descriptor individually with two threshold values. The comparison results were represented by three combinations: 11, 10, and 00. Correspondingly, a 256-bit BSIFT descriptor string was obtained. This approach improved matching accuracy to some extent; however, the matching time increased compared with the approach proposed by Chen et al.

The aforementioned BSIFT methods and their improved algorithms mostly ignore the problem of mirror reflection, which results in a significant increase in the mismatch rate for a mirror image pair. Guo [17] presented a mirror reflection invariant descriptor (MIFT), which was inspired by SIFT. However, the matching time of MIFT is comparable to that of SIFT.

To address these problems, this paper presents a new horizontal or vertical mirror reflection invariant binary descriptor named MBR-SIFT, in addition to a novel image matching approach. MBR-SIFT not only binarizes the SIFT descriptor, but also takes into consideration the problem of mirror reflection. First, 16 cells in the local region around the SIFT keypoint are reorganized, and then a 128-D vector of the SIFT descriptor is transformed into a reconstructed vector called R-SIFT according to eight directions. Finally, MBR-SIFT is obtained after R-SIFT binarization and reverse coding. To improve the matching speed and accuracy, a fast matching algorithm that includes a coarse-to-fine two-step matching strategy and two types of similarity measure for the MBR-SIFT descriptor are proposed. To examine the effectiveness of the proposed MBR-SIFT descriptor, it is also compared with other local descriptors.

## Related work

**Fig 1:**
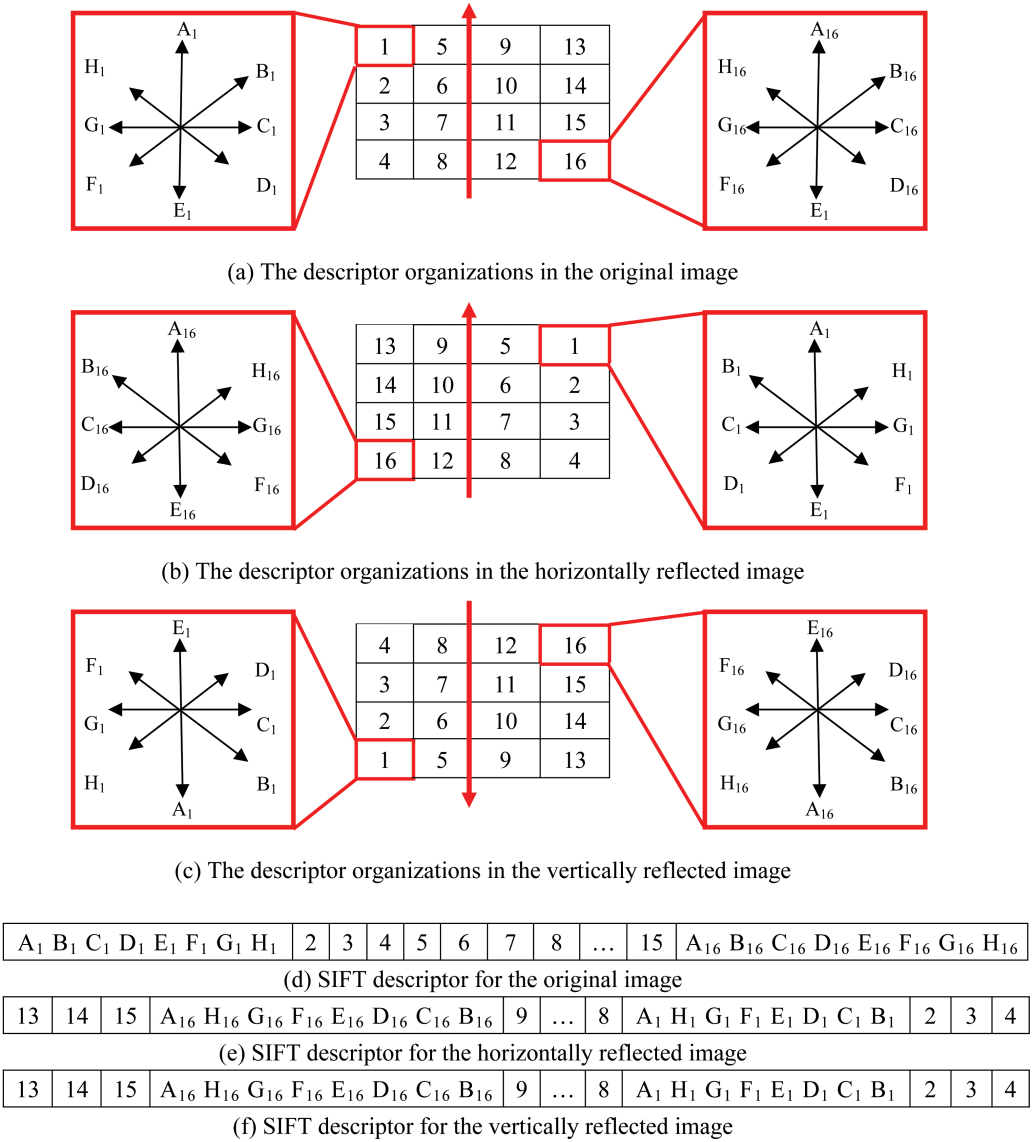
Illustration of the descriptor organization of SIFT with and without mirror reflection.

The SIFT algorithm extracts image features by searching the keypoints in the image, and then calculates the descriptors from the local region around the keypoints. As shown in Fig 1a, the local region is first divided into 16 cells with eight directions in each cell, and each direction is given a value. Finally, the 128-D SIFT descriptor, as shown in Fig 1d, isobtained.

The SIFT binarization approach is to transform the 128-D descriptor (*d*_0_, *d*_1_,…, *d*_127_) into a set of binary numeric strings. The commonly used binarization approaches can be classified into two common categories. The first category is proposed to compare the differential value *Ad*_*i*_ of the two adjacent values in a descriptor with the predefined threshold *M*:

$$Ad_{i}=\left\{ \begin{array}{l} \left|d_{i+1}-d_{i}\right|,\;if\;i<127\\ \left|d_{0}-d_{127}\right|,\;otherwise \end{array}\right.$$ (1)

$$b_{i}=\left\{ \begin{array}{l} 0,\;if\;Ad_{i}\leq M\\ 1,\;otherwise \end{array}\right.$$ (2)

The comparison result *b*_*i*_ is zero or one, which is only denoted by one bit [15].

The second category directly compares each *d*_*i*_ of the 128-D descriptor (*d*_0_, *d*_1_,…, *d*_127_) with two thresholds, *M*_1_ and *M*_2_:

$$b(i,i+128)=\left\{ \begin{array}{l} (1,1),\;if\;d_{i}>M_{1}\\ (1,0),\;if\;M_{2}<d_{i}\leq M_{1}\\ (0,0),\;if\;d_{i}\leq M_{2} \end{array}\right.$$ (3)

The comparison result is 11, 10, and 00, which is denoted by two bits [16]:

In essence, the first category converts the original 128 decimal values to a 128-bit binary value, which decreases the memory requirements and reduces the matching time. The problem of this type of approach is that it weakens the discriminative power of the SIFT descriptors. Regarding the discriminative power of SIFT descriptors, the second category is better than the first; however, its matching speed relative to the first category is slower.

The number of mismatched pairs would significantly increase for a mirror image pair, whether the matched features are SIFT descriptors or BSIFT descriptors. As shown in Fig 1b, once the local region around the keypoint is horizontally reflected, four columns comprised of 16 cells, in addition to eight directions in each cell are correspondingly horizontally reflected, and the corresponding 128-D SIFT descriptor is shown in Fig 1e. By comparing Fig 1e with Fig 1d, we can easily identify that the difference between both SIFT descriptors is large, which implies that SIFT is not horizontal mirror reflection invariant. Once the local region around the keypoint is vertically reflected, as shown in Fig 1f, the corresponding 128-D SIFT descriptor is the same as the scenario with the horizontal mirror reflection. Thus, SIFT is also not vertical mirror reflection invariant. Similarly, the binary SIFT descriptor is also not horizontal or vertical mirror reflection invariant.

## Our approach

An intuitive idea to make a BSIFT descriptor mirror reflection invariant is to artificially reflect one of the matching image pairs and perform image matching once again. This approach is simple; however, the time for matching is increased because of the repetitive execution of the SIFT algorithm and binarization operation. The BSIFT descriptor of the mirror reflection image can be achieved if we conduct a simple operation on the original BSIFT descriptor, which leads to savings in computational time. The proposed binarization method, MBR-SIFT, is based on this idea.

### SIFT descriptor reconstruction

By analyzing the structure of the SIFT descriptor, we found that the connection between the BSIFT descriptors before and after mirror reflection can be built by reconstructing the SIFT descriptor.

**Fig 2:**
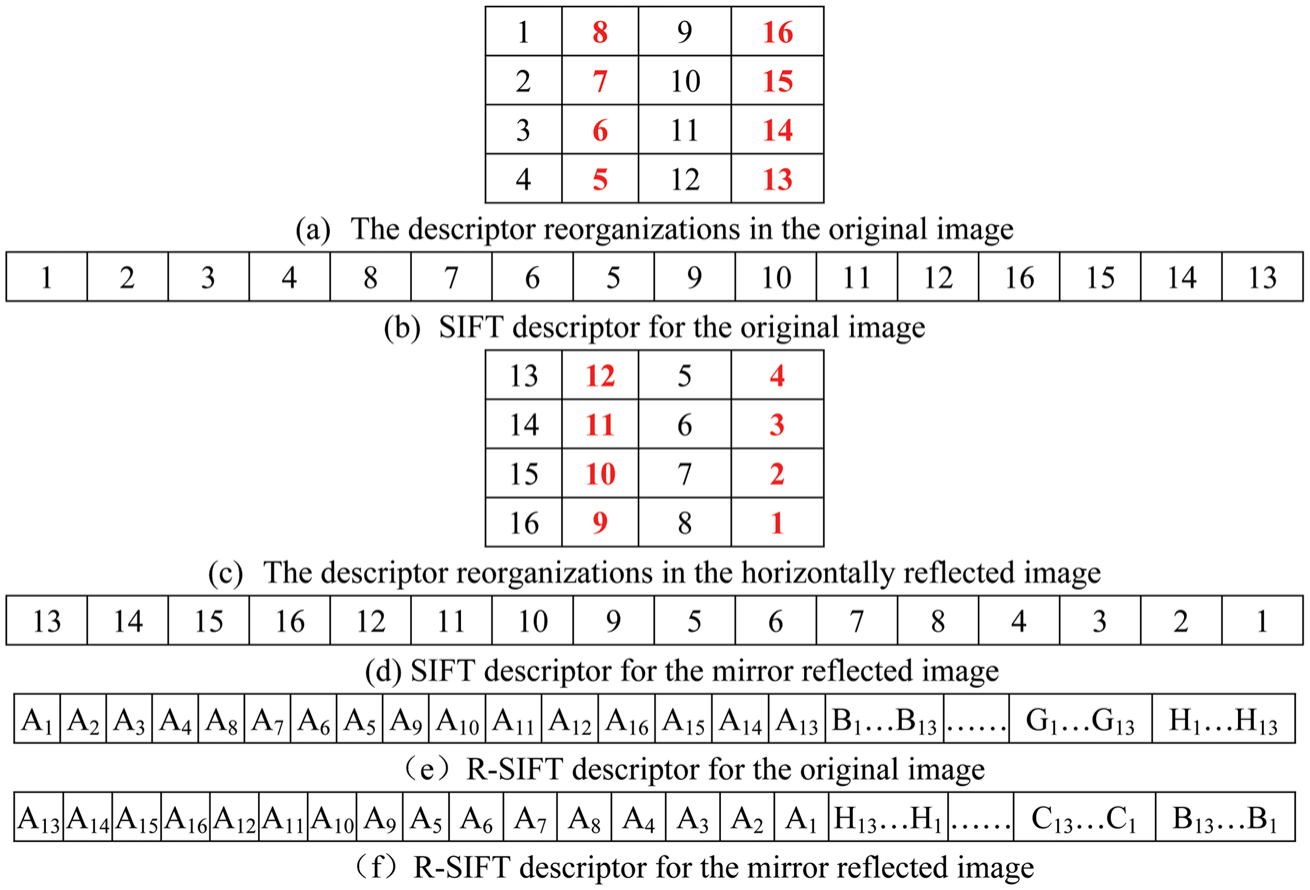
Illustration of the descriptor organization of R-SIFT with and without mirror reflection.

As shown in Fig 2a, the second and fourth columns of the 16 cells are reorganized in the reverse of their original order in Fig 1a, and correspondingly, in Fig 2b, the SIFT descriptor is reconstructed, in which the order of the 16 cells is “1,2,3,4,8,7,6,5,9,10,11,12,16,15,14,13.” Similarly, in Fig 2c and 2d, the order of the 16 cells in the horizontal or vertical mirror image is reconstructed as “13,14,15,16,12,11,10,9,5,6,7,8,4,3,2,1.” It can be observed that the order of the 16 cells for the image and its mirror image just meets the reversal relation. Additionally, each cell consists of eight oriented gradients, that is, “A_1_B_1_C_1_D_1_E_1_F_1_G_1_H_1_” for the first cell in Fig 1a and “A_1_H_1_G_1_F_1_E_1_D_1_C_1_B_1_” for the same cell after mirror reflection in Fig 1b and 1c. It can be observed that there is no reversal relation. To ensure the reversal relation, eight oriented gradients in 16 cells in Fig 2a are reorganized by their respective directions, and the reorganized SIFT descriptor, hereinafter referred to as the R-SIFT descriptor, is obtained, as shown in Fig 2e and 2f. The 128 elements of the original SIFT descriptor are organized by 16 cells and then eight directions. By contrast, the 128 elements of the R-SIFT descriptor are organized by eight directions and then 16 cells, which ensures the reversal relation between 16 elements in same direction for the original image and its mirror image.

### R-SIFT binarization

We denote the R-SIFT descriptor by the 128-D vector (*D*_0_, *D*_1_,…, *D*_127_) and the differential value by *AD*_*i*_(*i* = 0, 1,…, 127), given by

$$AD_{i}=\left\{ \begin{array}{l} D_{i+1}-D_{i}\;if\text{ mod}(i+1,16)\neq 0\\ D_{i-15}-D_{i}\;otherwise \end{array}\right.$$ (4)

The modulo operation in Eq (4) ensures that *AD*_*i*_ is the difference between adjacent values in the same direction.

Two SIFT binarization methods for *AD*_*i*_ are proposed to work with the following fast matching algorithm. The first is to compare *AD*_*i*_ with zero, and the comparison result is zero or one, only denoted by one bit. Thus, the 128-D R-SIFT descriptor is transformed into a 128-bit binary string denoted as BR-SIFT1: $\left\{b_{0}^{1},b_{1}^{1},\ldots ,b_{127}^{1}\right\}$. This procedure can be illustrated as follows:

$$b_{i}^{1}=\left\{ \begin{array}{l} 1\;if\;AD_{i}\geq 0\\ 0\;otherwise \end{array}\right.$$ (5)

The second SIFT binarization method is to compare *AD*_*i*_ with a threshold (±*T*), and the comparison result is 00, 01, 10, or 11, represented by two bits. Thus, the 128-D R-SIFT descriptor is transformed into a 256-bit binary string denoted as BR-SIFT2: $\left\{b_{0}^{2},b_{1}^{2},\ldots ,b_{254}^{2},b_{255}^{2}\right\}$. This procedure can be illustrated as follows:

$$(b_{2*i}^{2},b_{2*i+1}^{2})=\left\{ \begin{array}{l} \left(0,0)\;if\;AD_{i}\leq (-T\right)\\ (0,1)\;if\;(-T)<AD_{i}<0\\ (1,0)\;if\;0\leq AD_{i}<T\\ (1,1)\;if\;AD_{i}\geq T \end{array}\right.$$ (6)

where *T* is a positive value defined as

$$\bar{D}=\frac{\sum_{j=0}^{127}D_{j}}{128}$$ (7)

$$\sigma =\sqrt{\frac{\sum_{j=0}^{127}{\left(D_{j}-\bar{D}\right)}^{2}}{128}}$$ (8)

T=a×σ+b   (9)

where $\bar{D}$ and *σ* are the mean and standard deviation of the 128-D R-SIFT descriptor (*D*_0_, *D*_1_,…, *D*_127_), respectively; and *a* and *b* are constants. Through numerous experiments, the optimal *a* and *b* values were determined to be 2.3 and 0, respectively.

### Reverse coding

After constructing the BR-SIFT1 and BR-SIFT2 descriptors for the original image, we illustrate how to construct their corresponding descriptors for its horizontal and vertical mirror image denoted as MBR-SIFT1 and MBR-SIFT2, respectively.

From Table 1, it can be observed that the R-SIFT descriptors of the original image and its mirror image have a reversal relation. Thus, after the differential operation, with the exception that $b_{15}^{1}$ is changed into ~$b_{15}^{1}$, where ~ represents the NOT operator, the remaining bits in direction A of the BR-SIFT1 and MBR-SIFT1 descriptors are a mirror of each other. Regarding the first 15 bits in direction A, the MBR-SIFT1 descriptor can be recovered by scanning the BR-SIFT1 descriptor in reverse order and then performing the bitwise NOT operation. Additionally, the order of the eight directions in the BR-SIFT1 descriptor for the original image is “ABCDEFGH.” Despite this, the order of the eight directions in the MBR-SIFT1 descriptor for its mirror image is “AHGFEDCB;” therefore, we must exchange 16 binary values of directions B and H, directions C and G, and directions D and F in the BR-SIFT1 descriptor. Under this scheme, the MBR-SIFT1 descriptor is constructed from the BR-SIFT descriptor. Similarly, MBR-SIFT2 can also be constructed from BR-SIFT2; the difference is that the length of the descriptor for BR-SIFT2 is 256 bits. In Eq (6), it can be observed that if two differential values are symmetric with respect to the axis, their encoding value is also inverse, that is, (0,0) and (1,1), and (0,1) and (1,0). Therefore, in the procedure of the bitwise NOT operation, (0,0) and (1,1), and (0,1) and (1,0) are exchanged.

**Table 1:** Comparison of descriptors with or without reflection in the A direction.

original	R-SIFT descriptor	A_1_	A_2_	A_3_	A_4_	A_8_	A_7_	A_6_	A_5_	A_9_	A_10_	A_11_	A_12_	A_16_	A_15_	A_14_	A_13_
original	Differential value	A_2_- A_1_	A_3_- A_2_	A_4_- A_3_	A_8_- A_4_	A_7_- A_8_	A_6_- A_7_	A_5_- A_6_	A_9_- A_5_	A_10_- A_9_	A_11_- A_10_	A_12_- A_11_	A_16_- A_12_	A_15_- A_16_	A_14_- A_15_	A_13_- A_14_	**A**_**1**_**- A**_**13**_
original	BR-SIFT1 descriptor	*b*_0_^1^	*b*_1_^1^	*b*_2_^1^	*b*_3_^1^	*b*_4_^1^	*b*_5_^1^	*b*_6_^1^	*b*_7_^1^	*b*_8_^1^	*b*_9_^1^	*b*_10_^1^	*b*_11_^1^	*b*_12_^1^	*b*_13_^1^	*b*_14_^1^	***b***_**15**_^**1**^
mirror	R-SIFT descriptor	A_13_	A_14_	A_15_	A_16_	A_12_	A_11_	A_10_	A_9_	A_5_	A_6_	A_7_	A_8_	A_4_	A_3_	A_2_	A_1_
mirror	Differential value	A_14_- A_13_	A_15_- A_14_	A_16_- A_15_	A_12_- A_16_	A_11_- A_12_	A_10_- A_11_	A_9_- A_10_	A_9_- A_9_	A_6_- A_5_	A_7_- A_6_	A_8_- A_7_	A_4_- A_8_	A_3_- A_4_	A_2_- A_3_	A_1_- A_2_	**A**_**13**_**- A**_**1**_
mirror	MBR-SIFT1 descriptor	*~b*_14_^1^	*~b*_13_^1^	*~b*_12_^1^	*~b*_11_^1^	*~b*_10_^1^	*~b*_9_^1^	*~b*_8_^1^	*~b*_7_^1^	*~b*_6_^1^	*~b*_5_^1^	*~b*_4_^1^	*~b*_3_^1^	*~b*_2_^1^	*~b*_1_^1^	*~b*_0_^1^	***~b***_**15**_^**1**^

We compare the time complexity between the proposed MBR-SIFT method and original BSIFT method. Both the MBR-SIFT and BSIFT methods achieve 128-bit or 256-bit binary descriptors after the differential operation. Unlike BSIFT, to improve matching efficiency and accuracy, the MBR-SIFT method obtains two types of binary descriptors, BR-SIFT1 and BR-SIFT2, from the differential values. Instead of performing the SIFT algorithm on the mirror reflected image, the MBR-SIFT1 and MBR-SIFT2 binary descriptors for the mirror reflected image are directly constructed from BR-SIFT1 and BR-SIFT2, respectively, by inverse coding, which significantly reduces the computational time. The proposed binarization algorithm includes bitwise operations, such as binary digits exchange. The computational speed of MBR-SIFT is faster than that of SIFT and slightly lower than that of BSIFT.

## Two-step matching

To consider matching accuracy and computational efficiency, we present a coarse-to-fine two-step matching strategy. Coarse matching is performed based on the BR-SIFT1 and MBR-SIFT1 descriptors. The Hamming distance is used to measure the similarity between descriptors. The keypoints that correspond to the minimum distance are selected as the candidate keypoints for further use. Then, in the refining stage, the matching pair is selected from the candidate keypoints using the improved Hamming distance as the similarity measurement between the BR-SIFT2 and MBR-SIFT2 descriptors.

### Coarse matching

Suppose *I*_1_ and *I*_2_ are an image pair to be matched. For example, let $B_{1}^{1}$ denote the BR-SIFT1 descriptor of keypoint *a*_1_ in *I*_1_, and $B_{2}^{1}$ and $M_{2}^{1}$ denote the BR-SIFT1 and MBR-SIFT1 descriptors, respectively, of keypoint *a*_2_ in *I*_2_. Then calculate the Hamming distance between $B_{1}^{1}$ and $B_{2}^{1}$, and $B_{1}^{1}$ and $M_{2}^{1}$, and take the smaller value as the distance between keypoints *a*_1_ and *a*_2_. In coarse matching, the BR-SIFT1 descriptor of each keypoint in *I*_1_ is compared with that of both the BR-SIFT1 and MBR-SIFT1 descriptors in *I*_2_. Finally, the first *n* keypoints with the smallest distance in *I*_2_ are selected as the candidate keypoints, where *n* is computed as

$$n=\left\{ \begin{array}{l} 2\;if\;d_{\text{min}}<d_{\text{min}}^{\prime }*ratio\\ 5\;otherwise \end{array}\right.$$ (10)

where *d*_min_ and $d_{\text{min}}^{\prime }$ represent the smallest distance and second smallest distance, respectively, and *ratio* ∈ [0, 1] is a predefined threshold value. If *ratio* is equal to one, *n* has a higher probability of being two, while too few candidate keypoints affect the matching accuracy. By contrast, if *ratio* is equal to zero, *n* has a higher probability of being five, while too many candidate keypoints decrease the matching speed. Considering these two aspect of the matching problem, *ratio* is set to 0.5.

### Fine matching

We redefine the similarity measurement between two keypoints based on the Hamming distance. As shown in Algorithm 1, the new similarity measurement is designed for 256-bit BR-SIFT2 or MBR-SIFT2 descriptors. For these 256-bit binary descriptors, the Hamming distance is calculated once per four bits. If the result is zero, counter *counter*1 or *counter*2 is increased by one, which can further improve matching accuracy.

Algorithm 1: Similarity Measurement

Input: Image pair to be matched *I*_1_ and *I*_2_, BR-SIFT2 descriptor $\left\{b_{1,0}^{2},b_{1,1}^{2},\ldots ,b_{1,255}^{2}\right\}$ of SIFT keypoint *a*_1_ in *I*_1_, BR-SIFT2 binary descriptor $\left\{b_{2,0}^{2},b_{2,1}^{2},\ldots ,b_{2,255}^{2}\right\}$ and MBR-SIFT2 binary descriptor $\left\{m_{2,0}^{2},m_{2,1}^{2},\ldots ,m_{2,255}^{2}\right\}$ of candidate keypoint *a*_2_ in *I*_2_.

Output: Distance *D* between keypoints *a*_1_ and *a*_2_.

For *i* = 0 to 63

 $t_{1}=\{b_{1,i*4}^{2},b_{1,i*4+1}^{2},b_{1,i*4+2}^{2},b_{1,i*4+3}^{2}\}$

 $t_{2}=\{b_{2,i*4}^{2},b_{2,i*4+1}^{2},b_{2,i*4+2}^{2},b_{2,i*4+3}^{2}\}$

 $t_{3}=\{m_{2,i*4}^{2},m_{2,i*4+1}^{2},m_{2,i*4+2}^{2},m_{2,i*4+3}^{2}\}$

 If Hamming(*t*_1_, *t*_2_) = 0

  *counter*1 = *counter*1 + 1

 If Hamming(*t*_1_, *t*_3_) = 0

  *counter*2 = *counter*2 + 1

End For

If *counter*2 > *counter*1

 *counter = counter*2

else *counter = counter*1

*D* = arccos(*counter*/64)

In fine matching, the BR-SIFT2 descriptor of each keypoint in *I*_1_ is compared with the corresponding *n* candidates in *I*_2_ and then we determine the matching pairs according to

$$match\;or\;not?\left\{ \begin{array}{ll} match & if\;vals(1)<vals(2)*distratio\\ not\;match & otherwise \end{array}\right.$$ (11)

where *vals*(1) and *vals*(2) represent the smallest distance and second smallest distance, respectively. Additionally, the predefined threshold *distratio* is empirically set to 0.84 in the experiment.

## Experiment

We evaluated the proposed approach, MBR-SIFT, on a public dataset, the UKBench dataset [18], which contains 10,200 images from 2,550 object/scene groups. Each group consists of four images taken from different views or in different imaging conditions.

To demonstrate the effectiveness of the improved similarity measurement, we also implemented another version called MBR-SIFT’, which still used the Hamming distance instead of the improved Hamming distance for fine matching. Moreover, we also implemented the original SIFT, and Chen’s [15] and Zhou’s [16] methods to compare accuracy and efficiency. Moreover, several local binary features, such as CS-LBP [19], BRIEF [20], BRISK[21], and FREAK[22], have been proposed recently with promising performance in image matching, and we also implemented them to compare their potential with BSIFT in image matching. All of these methods used Eq (11) to determine whether the keypoint pairs were matched.

The results are presented with recall versus *1-precision* [11,12] given by

$$recall=\frac{tn}{en}$$ (12)

$$1-precision=\frac{fn}{qn}$$ (13)

where *tn* and *en* represent the number of correct matches and ground truth number of matches between the images, respectively, and *fn* and *qn* represent the number of false matches and total number of matches between the images, respectively. To evaluate the performance of the image matching method, we need to determine matching pairs as much as possible with high accuracy [23], that is, when *1-precision* is the same, the performance for the method with a higher recall is better.

### Mirror reflection

**Fig 3:**
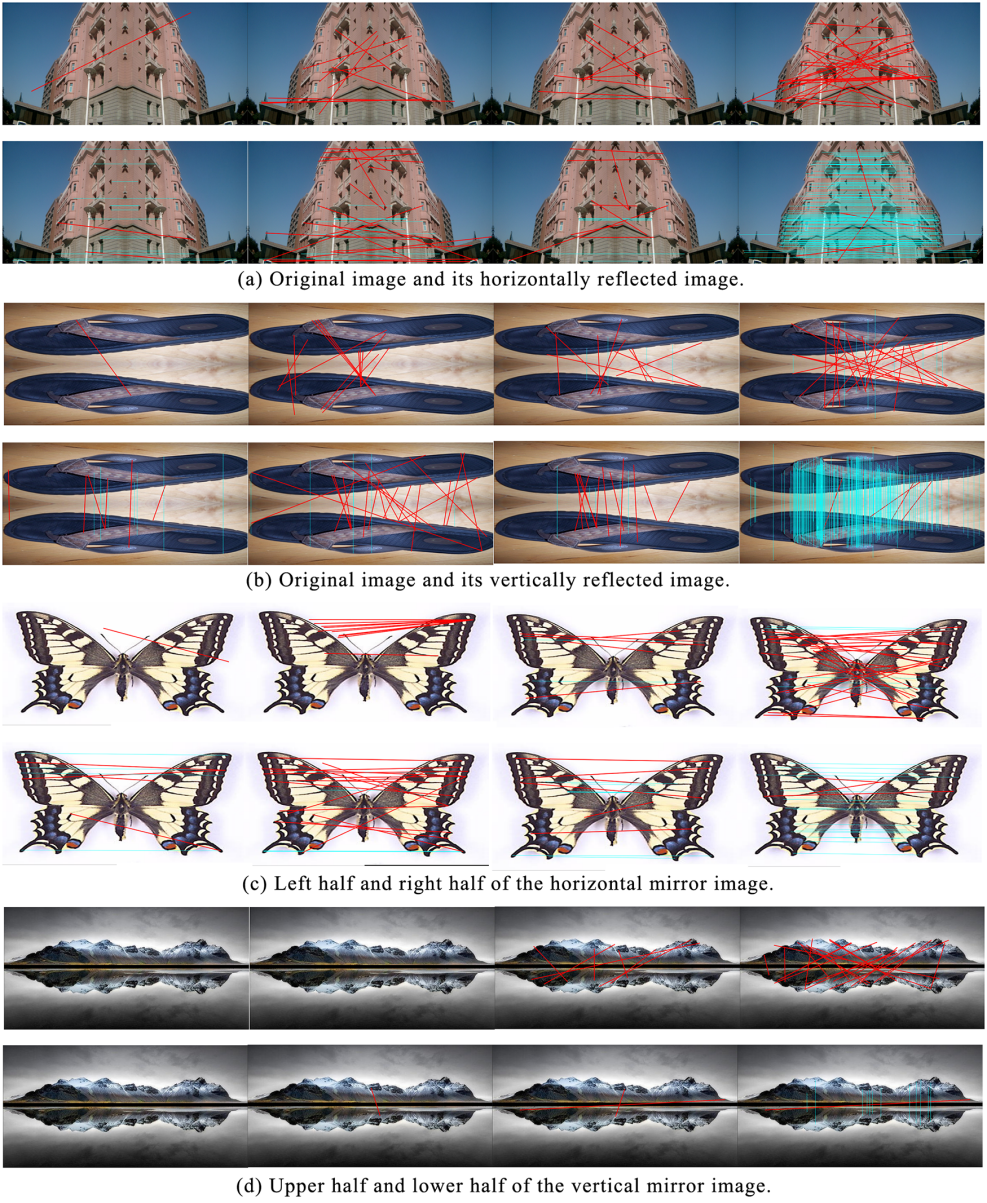
Comparing the matching performance of CS-LBP, BRIEF, BRISK, FREAK, SIFT, Chen’s method, Zhou’s method, and MBR-SIFT under mirror reflected transformation.

Fig 3 contrasts the matching performance of CS-LBP, BRIEF, BRISK, FREAK, SIFT, Chen’s method, Zhou’s method, and MBR-SIFT for images that had undergone reflection transformations (the first row in Fig 3a–3d shows the matching results of the first four methods, and the second row shows the matching results of the latter four methods), in which the blue lines and red thick lines represent the correct matches and false matches, respectively. Table 2 shows the matching results over the mirror reflection. It can be observed from Table 2 that the performance of MBR-SIFT is superior to the other methods in terms of accuracy and recall. Additionally, the image pairs of Fig 3a and 3b were generated from an artificial reflection. By contrast, the image pairs of Fig 3c and 3d originated from a mirror image, which implies lower similarity. Therefore, in terms of MBR-SIFT, the recall of Fig 3a and 3b is much higher than that of Fig 3c and 3d. In terms of CS-LBP and BRIEF, the accuracy and recall of Fig 3a–3d are zero, which demonstrates the worst performance. The main reason is that both CS-LBP and BRIEF are not robust to rotation and scaling.

**Table 2:** Matching results over mirror reflection.

Image pair	Method	# of feature points (left/right) or (up/ down)	# of matches	# of false matches	Accuracy (%)	Recall (%)
(a)	CS-LBP	200/289	1	1	0	0
	BRIEF	196/287	10	10	0	0
	BRISK	255/253	10	10	0	0
	FREAK	301/296	33	31	6.06	0.67
	SIFT	1051/1072	18	2	88.89	1.52
	Chen’s	1051/1072	36	22	38.89	1.33
	Zhou’s	1051/1072	15	9	40.00	0.57
	MBR-SIFT	1051/1072	214	5	97.66	19.90
(b)	CS-LBP	126/122	1	1	0	0
	BRIEF	125/121	13	13	0	0
	BRISK	138/133	14	10	28.57	3.01
	FREAK	151/148	35	25	28.57	6.76
	SIFT	926/900	15	6	60.00	1.00
	Chen’s	926/900	27	21	22.22	0.67
	Zhou’s	926/900	17	12	29.41	0.56
	MBR-SIFT	926/900	367	4	98.91	40.33
(c)	CS-LBP	141/153	1	1	0	0
	BRIEF	131/148	11	11	0	0
	BRISK	145/150	7	5	28.57	1.38
	FREAK	215/225	30	25	16.67	2.33
	SIFT	350/334	8	4	50.00	1.19
	Chen’s	350/334	21	16	23.81	1.50
	Zhou’s	350/334	13	7	46.15	1.80
	MBR-SIFT	350/334	23	3	86.96	5.99
(d)	CS-LBP	31/37	0	0	0	0
	BRIEF	30/36	0	0	0	0
	BRISK	101/89	7	7	0	0
	FREAK	156/162	24	24	0	0
	SIFT	225/354	0	0	0	0
	Chen’s	225/354	1	1	0	0
	Zhou’s	225/354	2	2	0	0
	MBR-SIFT	225/354	13	1	92.31	5.33

### Matching accuracy and efficiency

**Fig 4:**
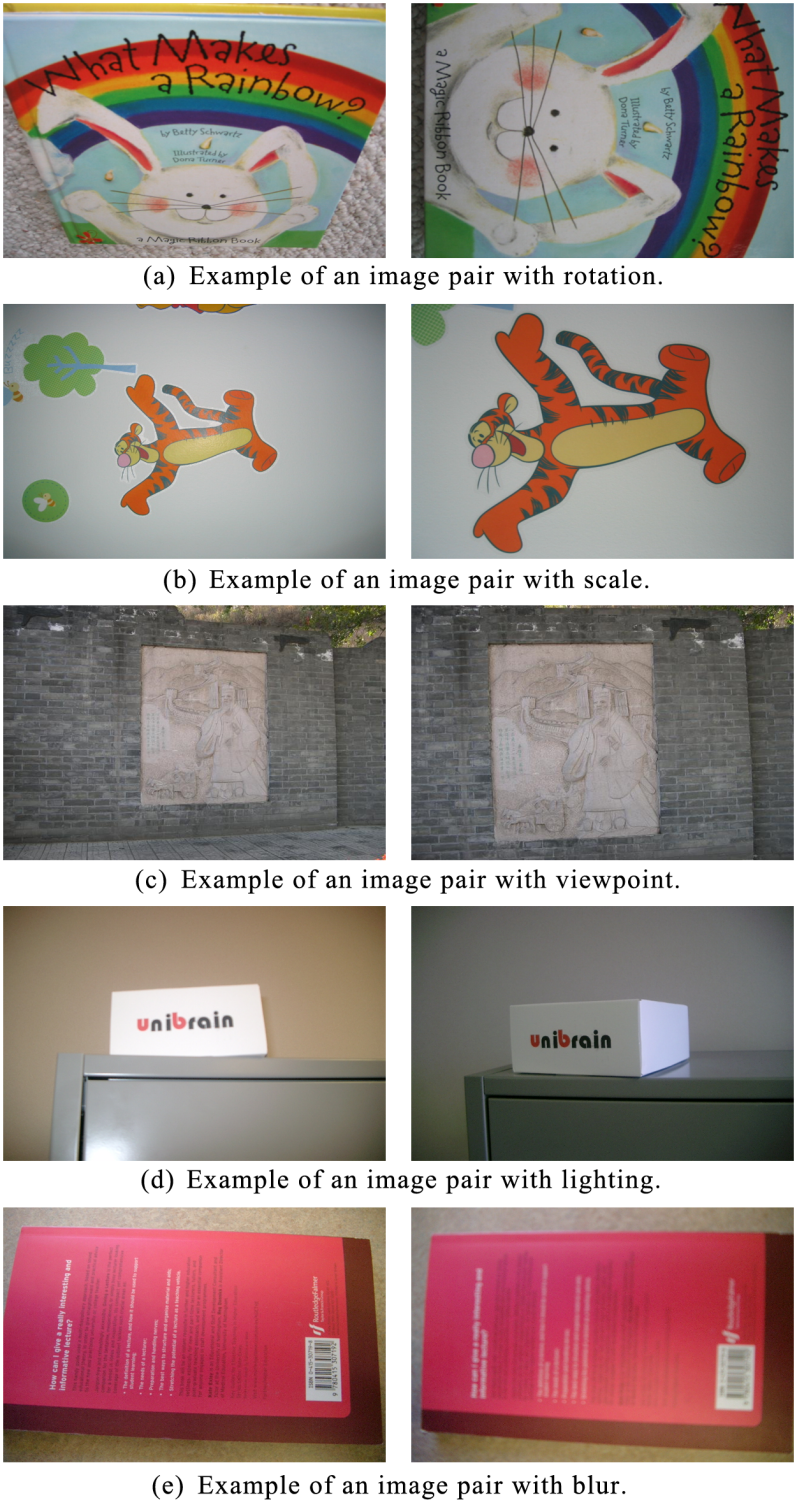
Examples of image pairs.

To evaluate the accuracy performance of MBR-SIFT, the nine methods, CS-LBP, BRIEF, BRISK, FREAK, SIFT, Chen’s method, Zhou’s method, MBR-SIFT’, and MBR-SIFT, were used in the matching experiments. We randomly selected 200 image pairs for the matching experiments. Some examples of image pairs with rotation, scale, viewport, lighting, and blur variance from the UKBench dataset are shown in Fig 4.

**Fig 5:**
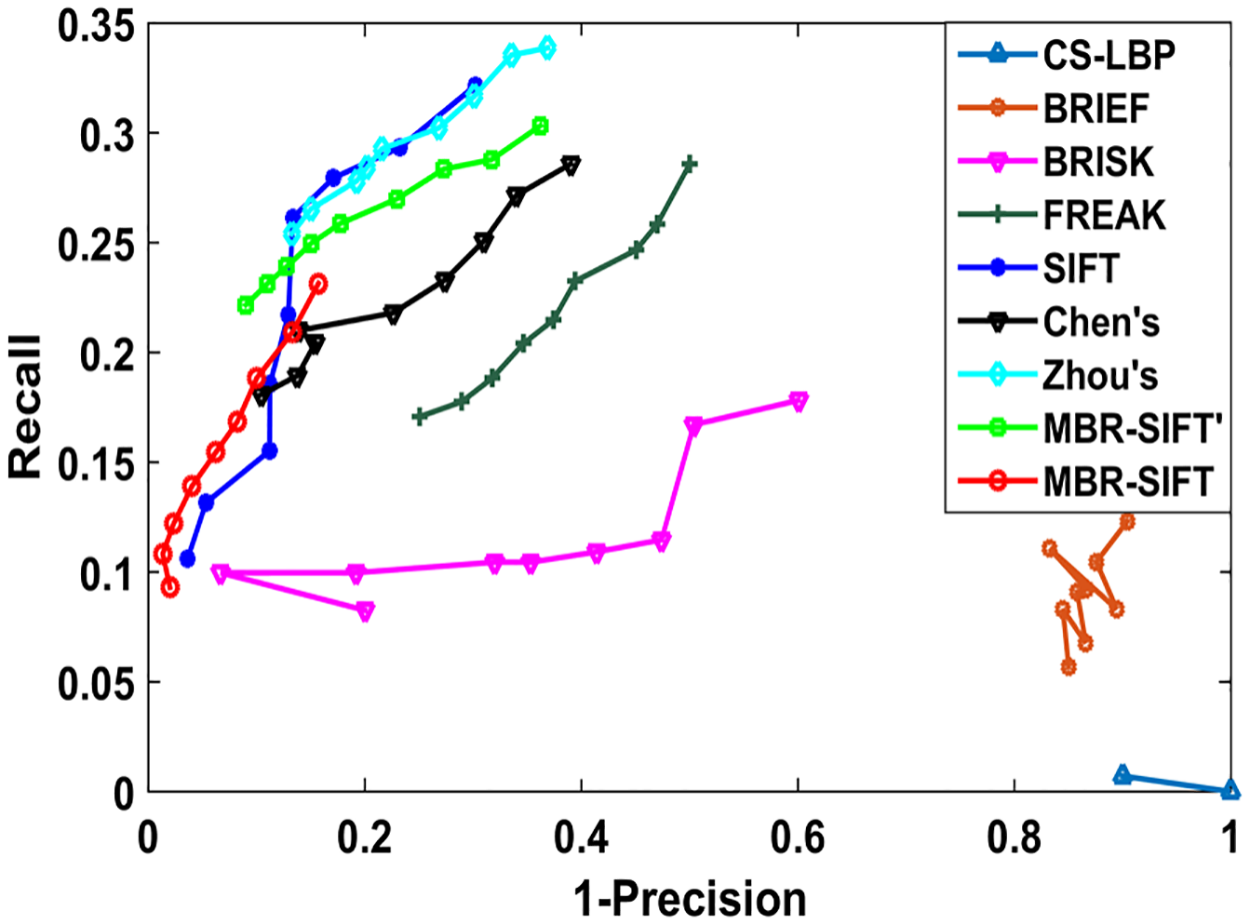
Recall versus 1-precision for nine methods.

We calculated recall and *1-precision* under different values of *distratio* for the several image pairs. With the threshold *distratio* ranging from 0.45 to 0.85, with an interval of 0.05 for the SIFT method, and from 0.72 to 0.88 with an interval of 0.02 for the remaining methods, we achieved the average results shown in Fig 5.

First, we compared five methods: SIFT, Chen’s method, Zhou’s method, MBR-SIFT’, and MBR-SIFT. Compared with the other methods, it can be observed from Fig 5 that MBR-SIFT achieved the highest matching accuracy. This is mainly because the improved distance measurement was used, which led to a stricter matching criteria than that of the other methods, and thus achieved higher matching accuracy. Despite this, recall was slightly decreased compared with other methods. However, matching accuracy is more important than recall in image matching.

Moreover, we also compared CS-LBP, BRIEF, BRISK, and FREAK with SIFT in addition to its variants. As shown in Fig 5, all of the four methods, CS-LBP, BRIEF, BRISK, and FREAK, performed much worse than SIFT and its variants in accuracy and recall. This is mainly because their discriminative power is not as good as SIFT and its variant feature BSIFT.

As shown in Table 3, the efficiency of the SIFT method was the lowest among the nine methods. Regarding MBR-SIFT, its computational time was approximately the same as that of MBR-SIFT’, slightly higher than that of Chen’s method, and lower than that of Zhou’s method. This is because Chen’s and Zhou’s methods generated 128-bit and 256-bit binary descriptors, respectively, whereas MBR-SIFT and MBR-SIFT’ generated binary descriptors that included both 128 bits and 256 bits. The efficiency of the other four methods, CS-LBP, BRIEF, BRISK, and FREAK, was higher than that of SIFT and its variants. This is because the number of features extracted by the four methods was significantly lower than that of SIFT and its variants.

**Table 3:** Performance for the nine methods.

-	CS-LBP	BRIEF	BRISK	FREAK	SIFT	Chen’s	Zhou’s	MBR-SIFT’	MBR-SIFT
Avg. matching time for an image(s)	7.89	1.20	4.09	5.86	34.72	13.42	23.15	15.88	15.91
Matching speedup ratio(relative to SIFT)	4.40	28.93	8.49	5.92	1	2.59	1.50	2.19	2.18

### Parameter analysis

Next, we conducted the experiments on 200 image pairs to investigate the impact of the number of candidate keypoints *n* on efficiency and accuracy.

**Fig 6:**
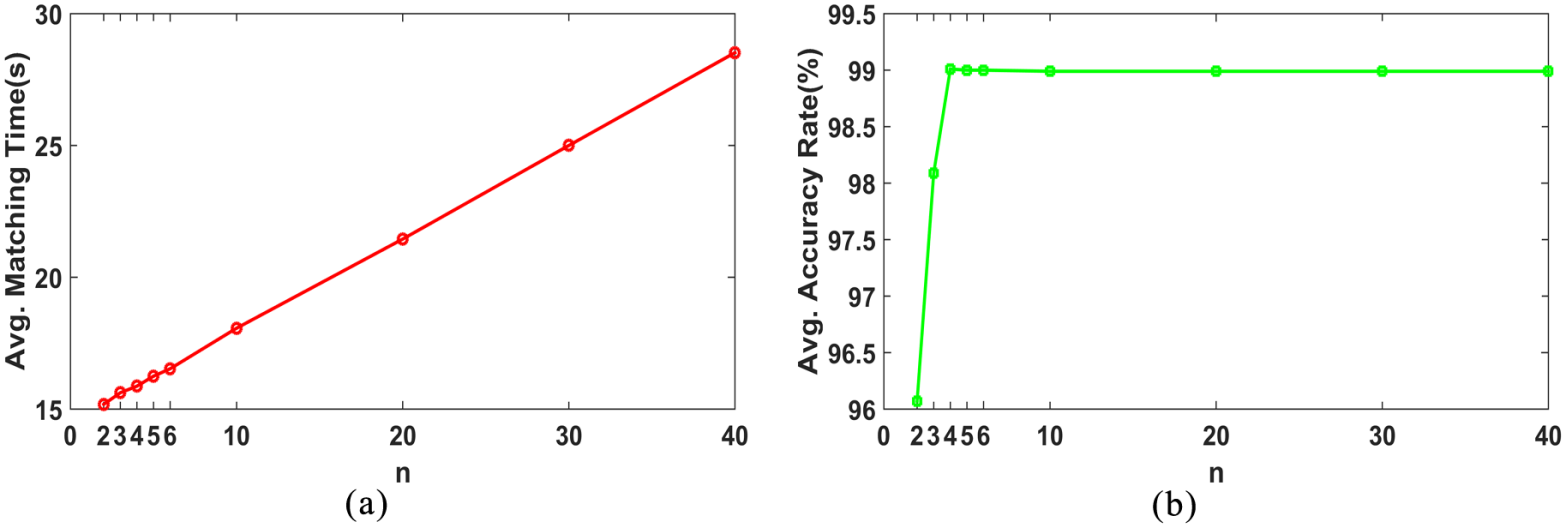
*n* vs matching time and matching accuracy.

For convenience, we ignored the criteria in Eq (10), where *n* is set in the range (0,40). In terms of efficiency, as shown in Fig 6a, the matching time increased as *n* increased. It can be observed from Fig 6b that the accuracy first increased as *n* increased to four, and then remained stable as *n* continued to increase. This implies that the candidate keypoints through coarse matching contained the correct matching pairs when *n* was greater than a certain value. In considering both matching time and accuracy, the maximum of *n* was set to five.

**Fig 7:**
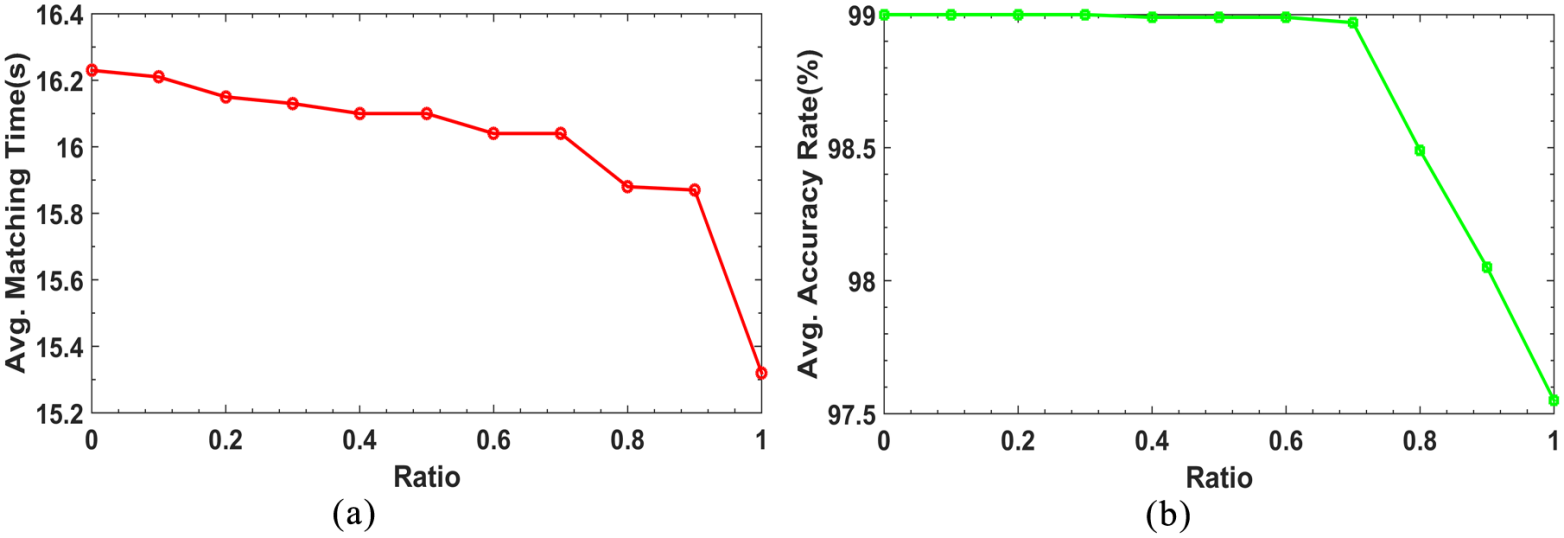
*Ratio* vs matching time and matching accuracy.

To improve matching efficiency, *n* is equal to either two or five in Eq (10). The value of *n* is determined by *ratio*. Fig 7a and 7b show the matching time and accuracy for different ratios, respectively. It can be seen from Fig 7a and 7b that both the matching time and accuracy decreased as the ratio increased, that is, the smaller the value of *ratio*, the longer the matching time and the higher the matching accuracy, and vice versa. *Ratio* is in the interval [0,1]. When *ratio* was set to zero, the value of *n* was five. When *ratio* was set to one, the value of *n* was two. This implies that the value of *ratio* only affected the matching time and accuracy for *n* between two and five. Therefore, in considering both the matching time and accuracy, *ratio* was set to 0.5.

## Conclusion

In this paper, we presented a binary SIFT descriptor (MBR-SIFT), which was achieved by reconstructing the SIFT descriptor. The MBR-SIFT descriptor is invariant to mirror reflection while being robust to rotation, scaling, viewpoint, lighting, and blur changes. Additionally, we also presented a coarse-to-fine two-step matching strategy, in addition to a novel similarity measure to further improve the performance of image matching. The experimental results show that the proposed method can achieve higher matching accuracy, whereas recall is slightly lower. In future research, we will consider how to ensure both high accuracy and recall.

## Supporting information

S1 Fig Comparing the matching performance for CS-LBP, BRIEF, BRISK, FREAK, SIFT, Chen’s method, Zhou’s method, MBR-SIFT’ and MBR-SIFT. (DOCX)
