# Supplementary material for: MBR-SIFT: A mirror reflected invariant feature descriptor using a binary representation for image matching
Source: PLoS One. 2017 May 18;12(5):e0178090. doi: 10.1371/journal.pone.0178090 (PMC5436860; doi:10.1371/journal.pone.0178090)
Supplement: S1 Fig — (DOCX) [file pone.0178090.s001.docx]

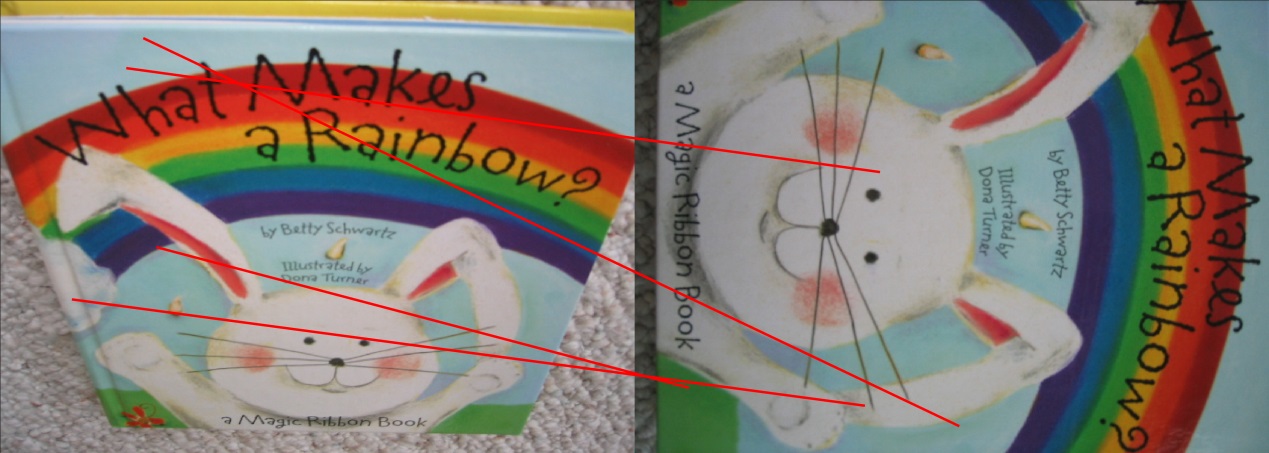


(a) CS-LBP


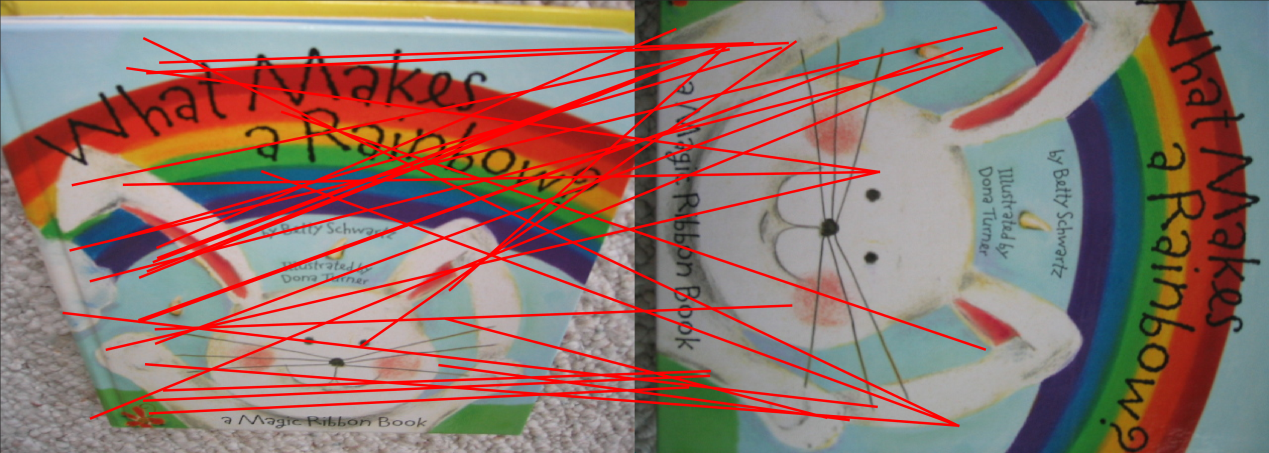


(a) BRIEF


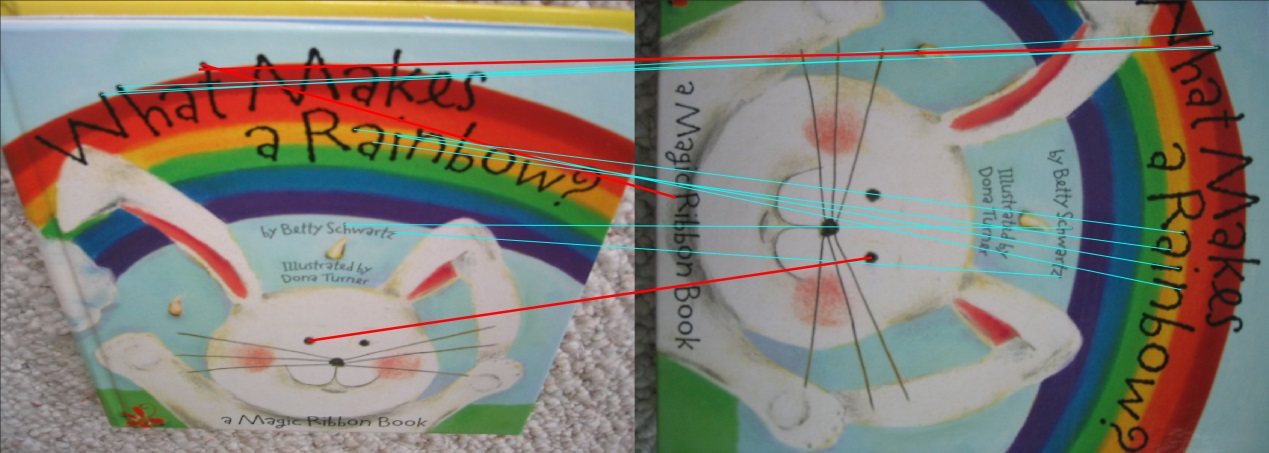


(a) BRISK
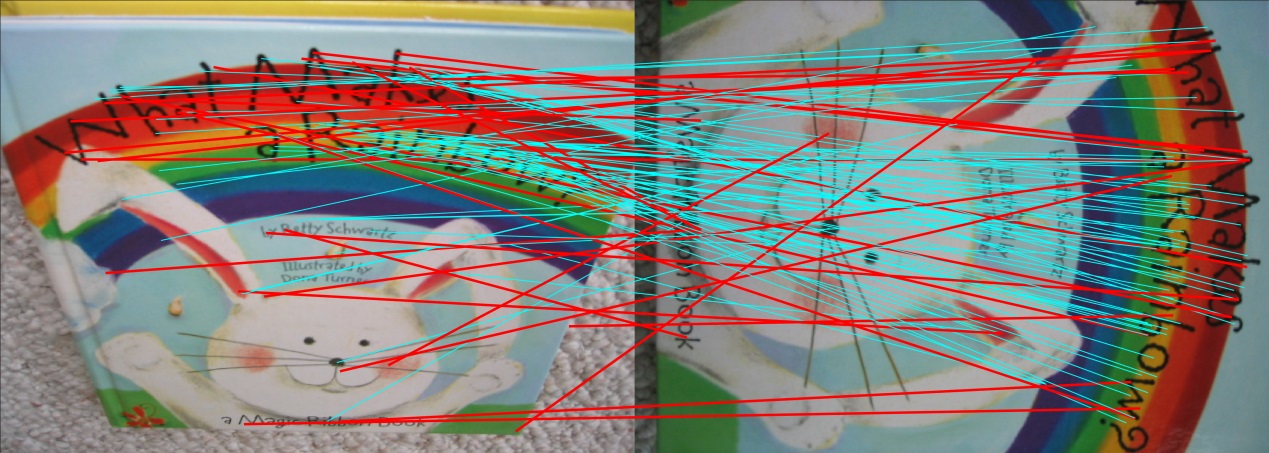


(a) FREAK


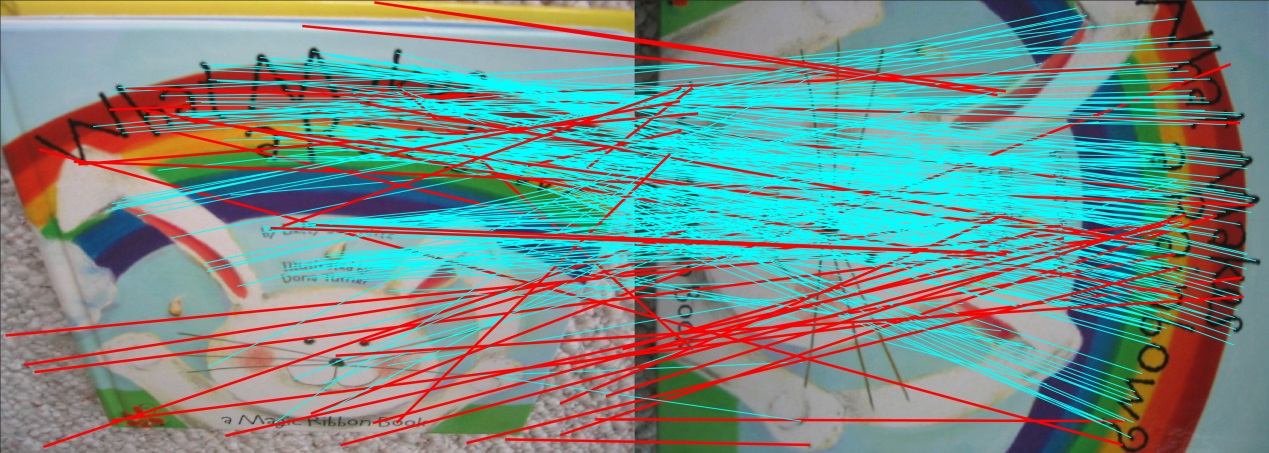


(a) SIFT


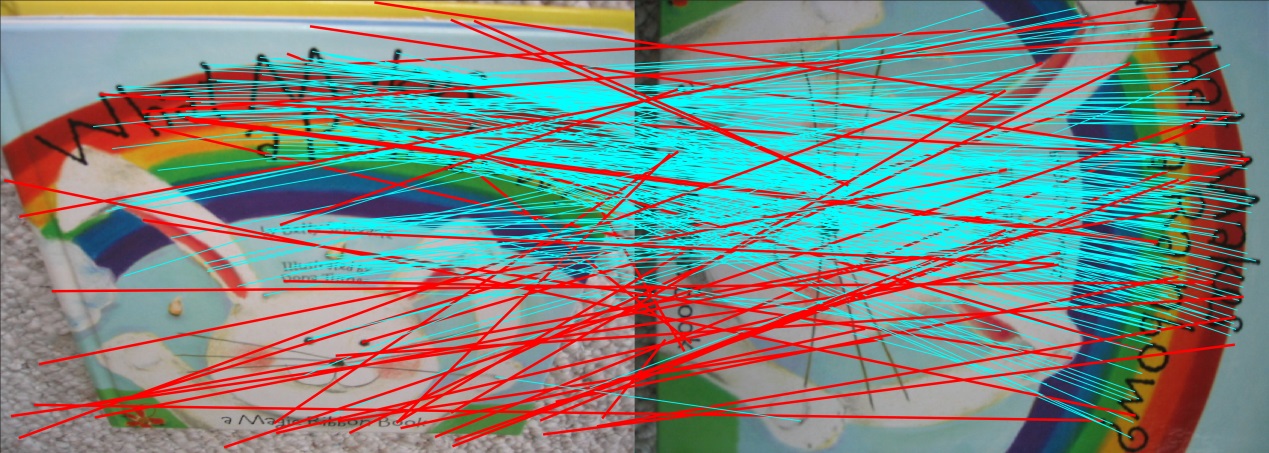


(a) Chen’s method


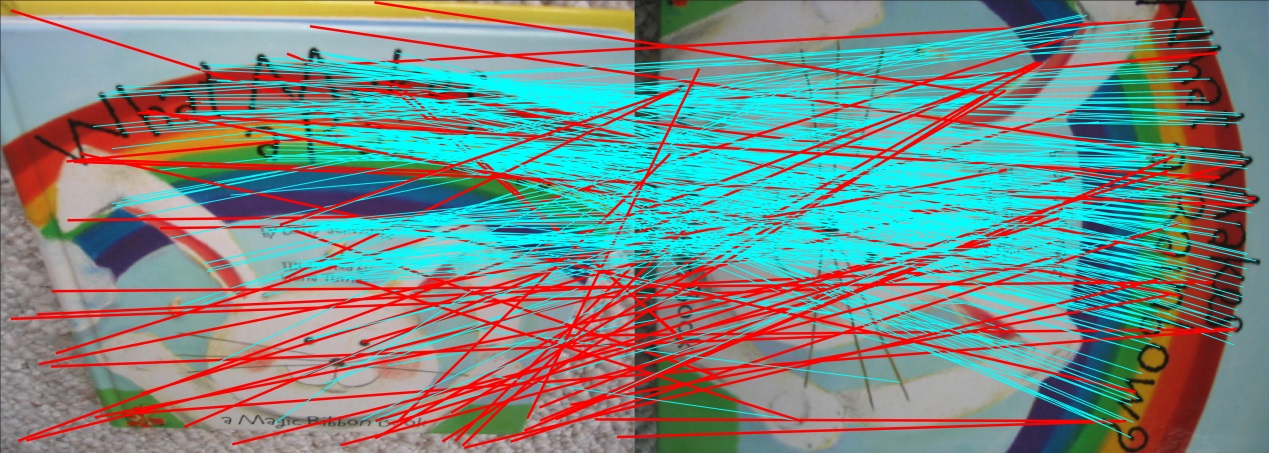


(a) Zhou’s method


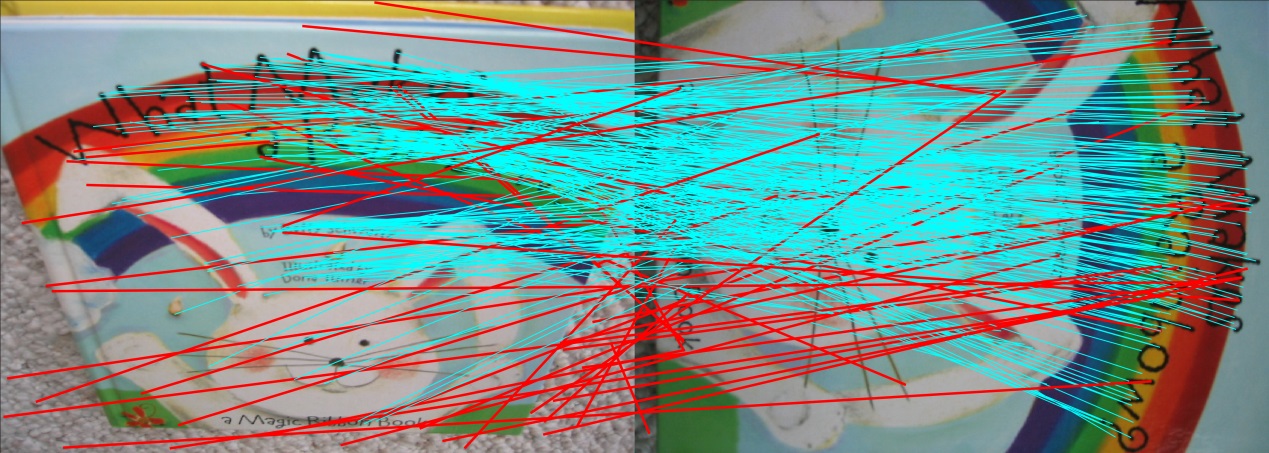


(a) MBR-SIFT’


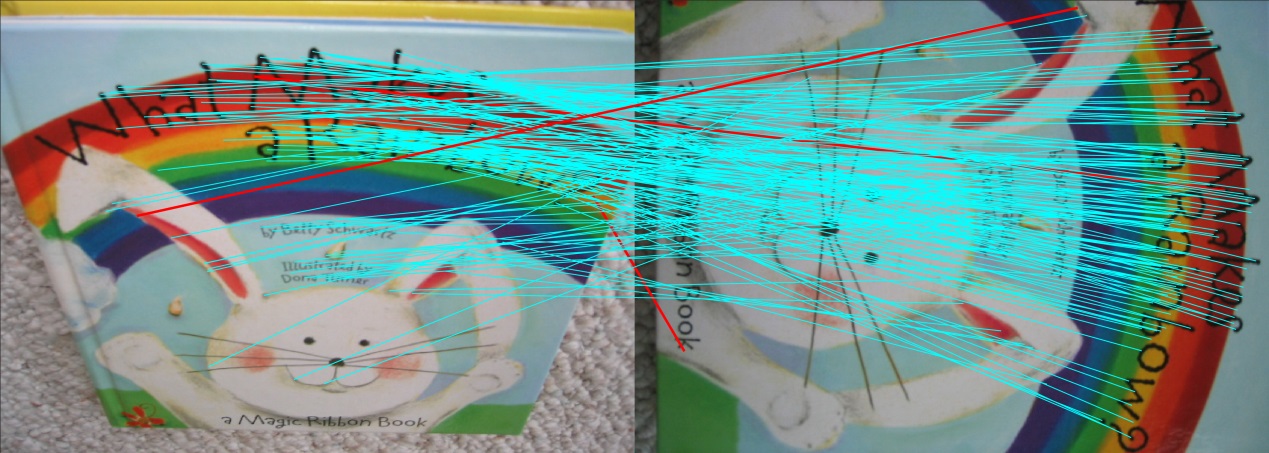


(a) MBR-SIFT


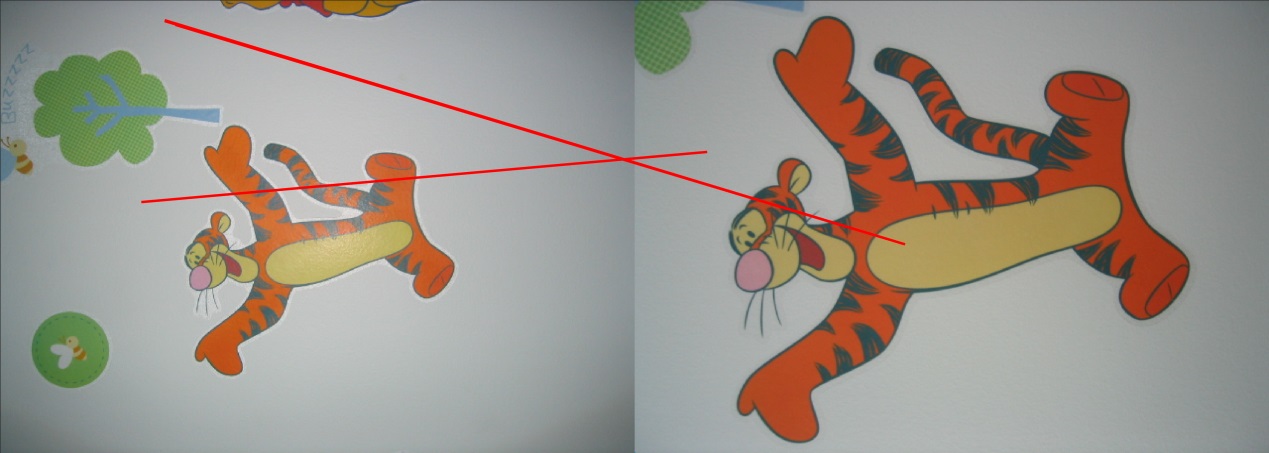


(b) CS-LBP
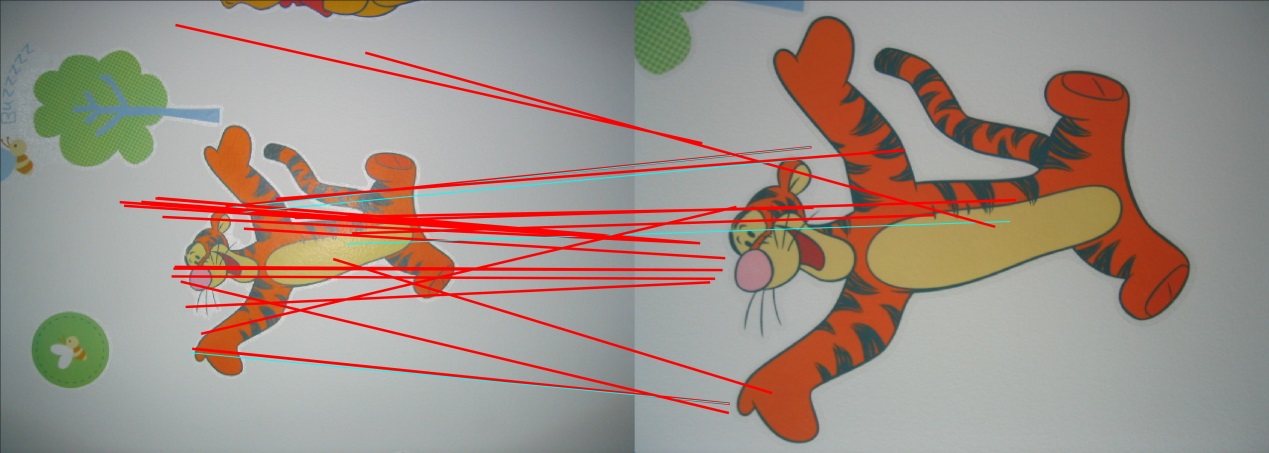


(b) BRIEF


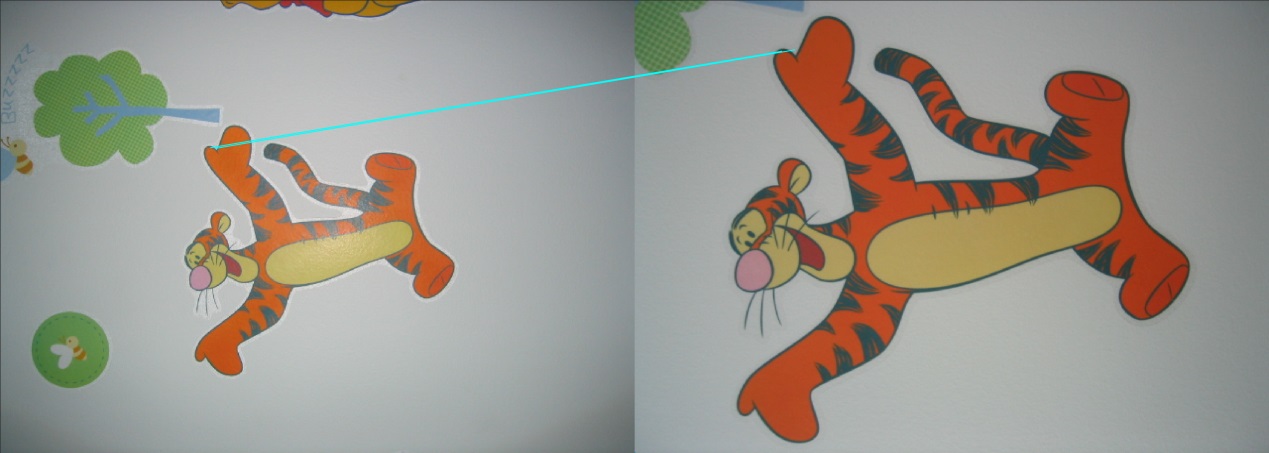


(b) BRISK


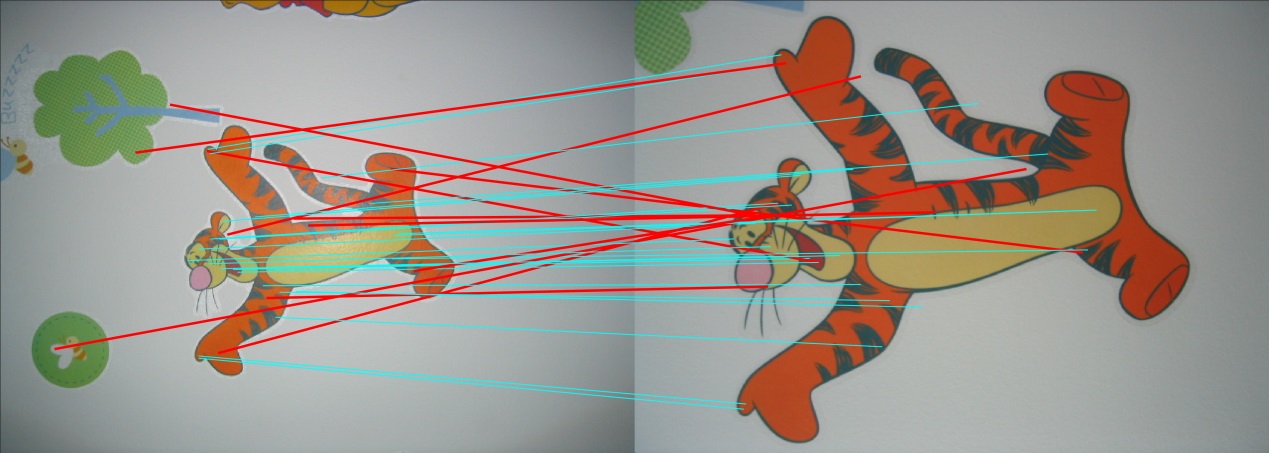


(b) FREAK


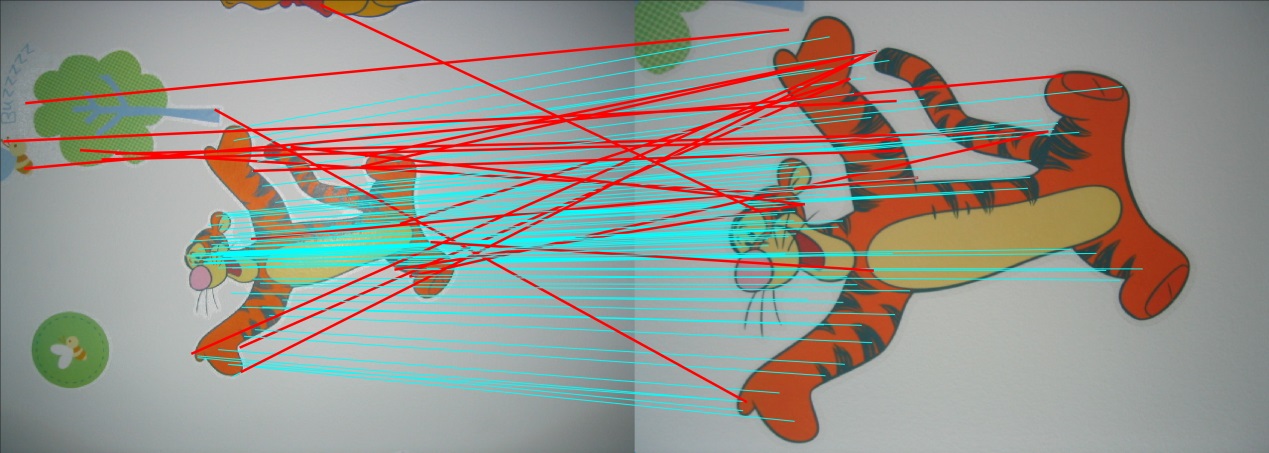


(b) SIFT


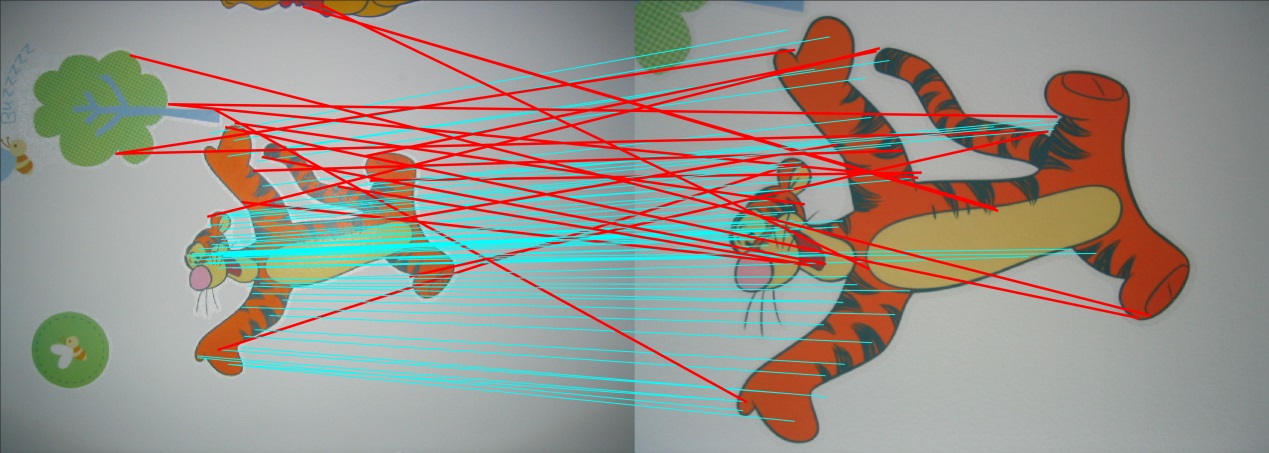


(b) Chen’s method


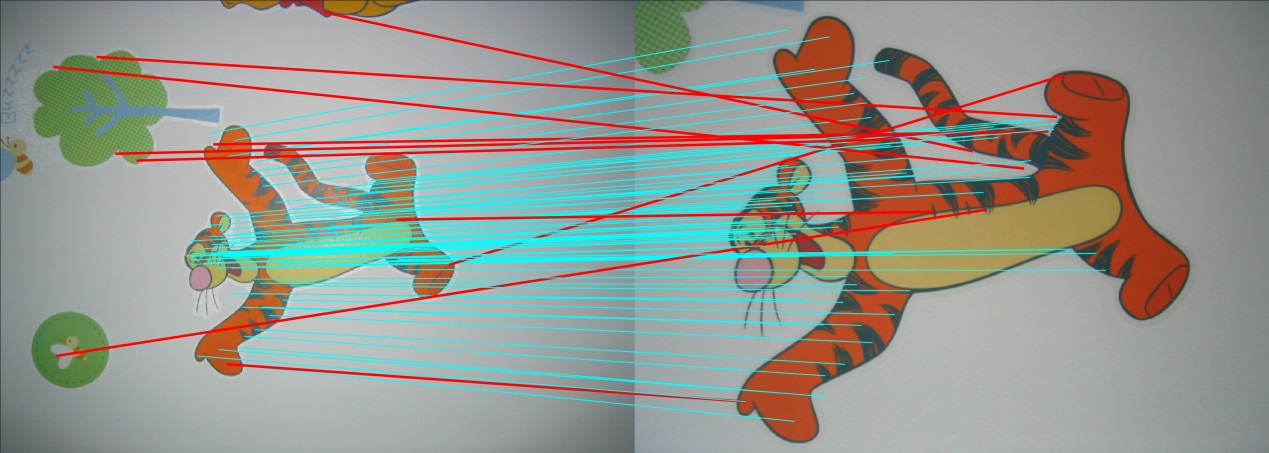


(b) Zhou’s method


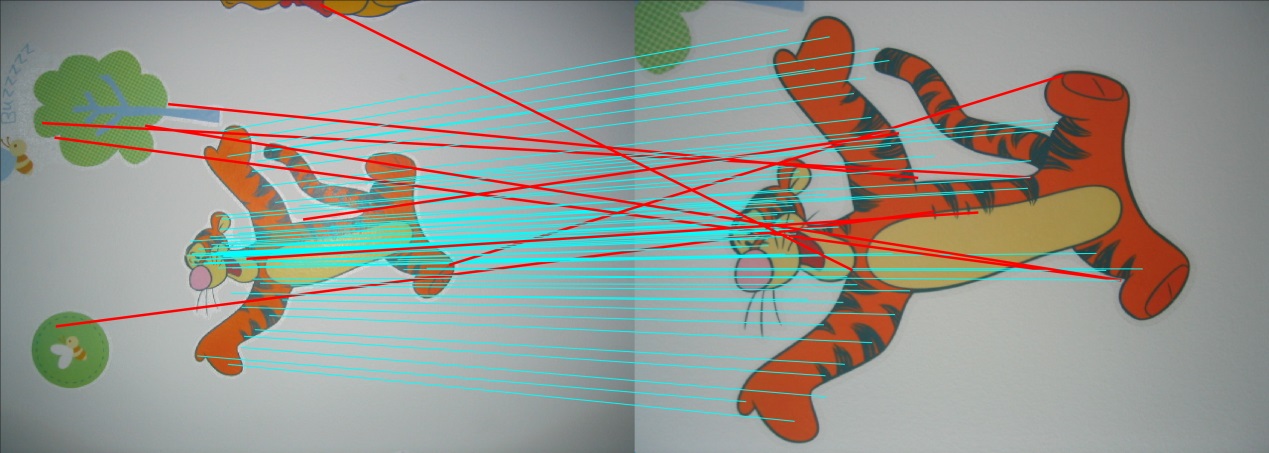


(b) MBR-SIFT’


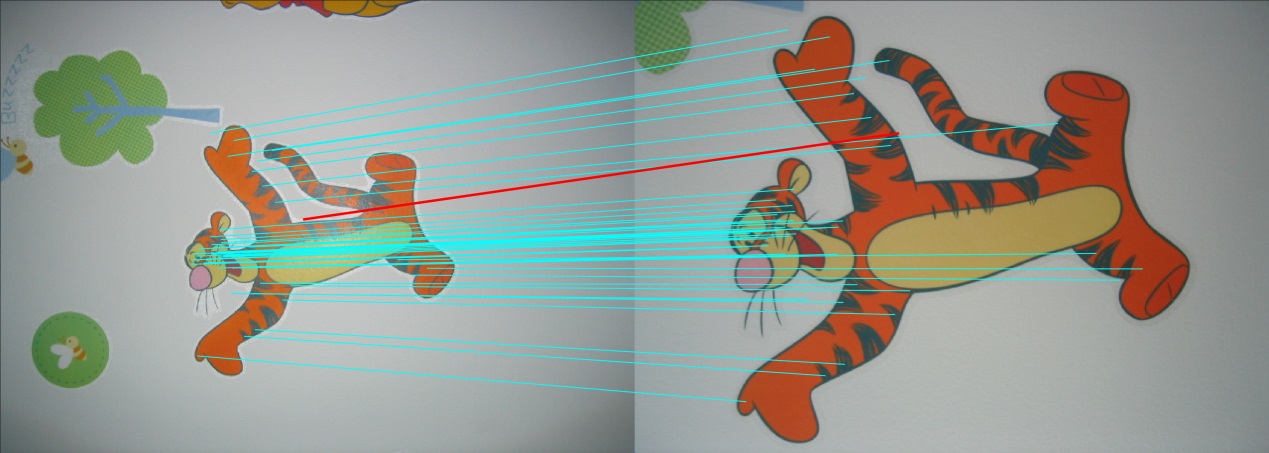


(b) MBR-SIFT


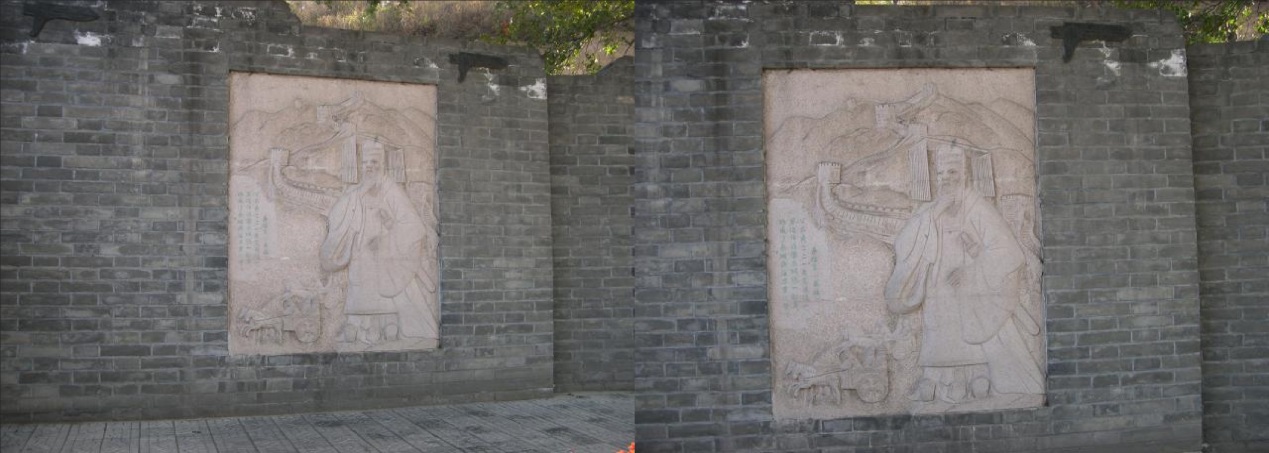


(c) CS-LBP
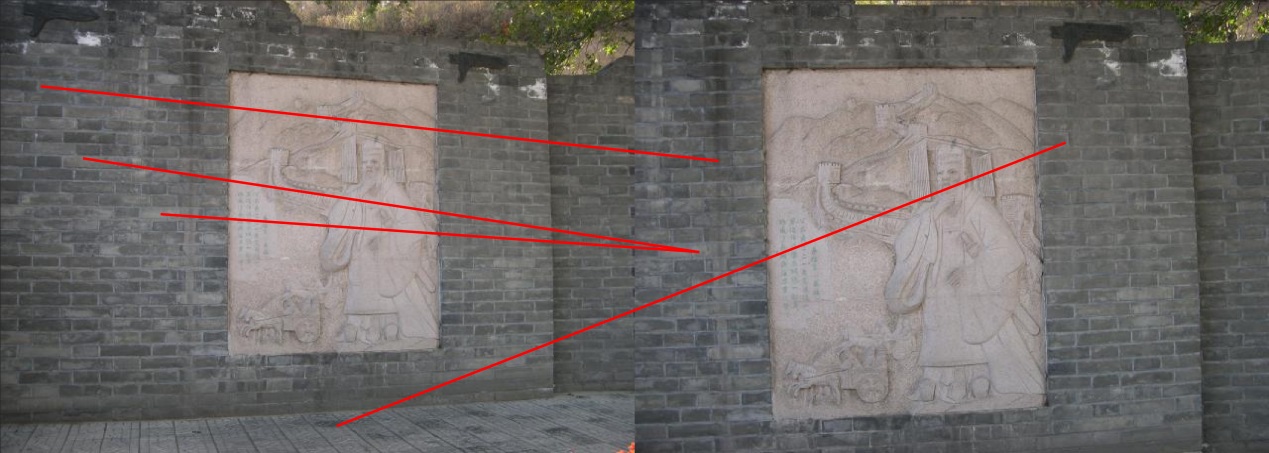


(c) BRIEF


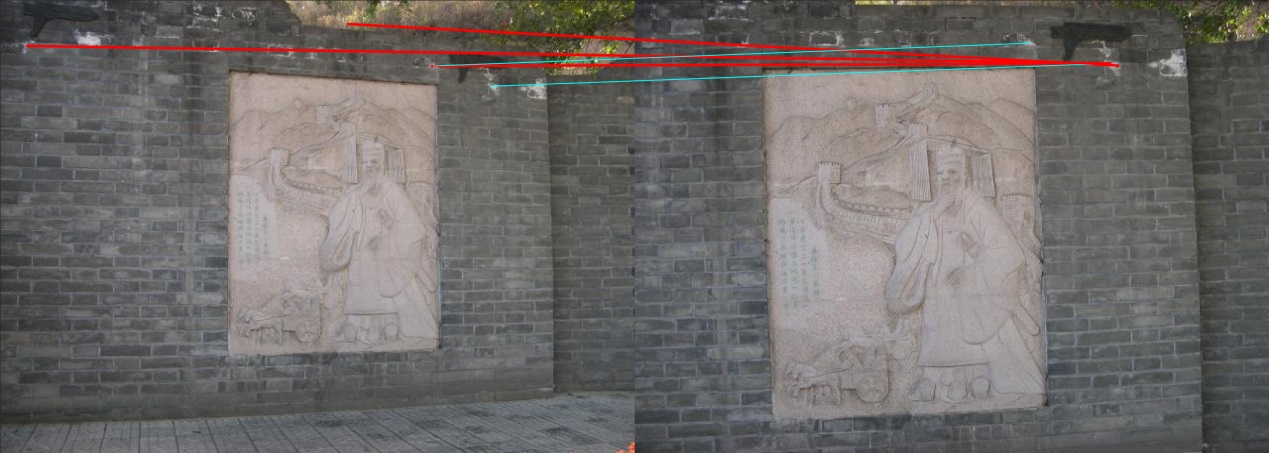


(c) BRISK


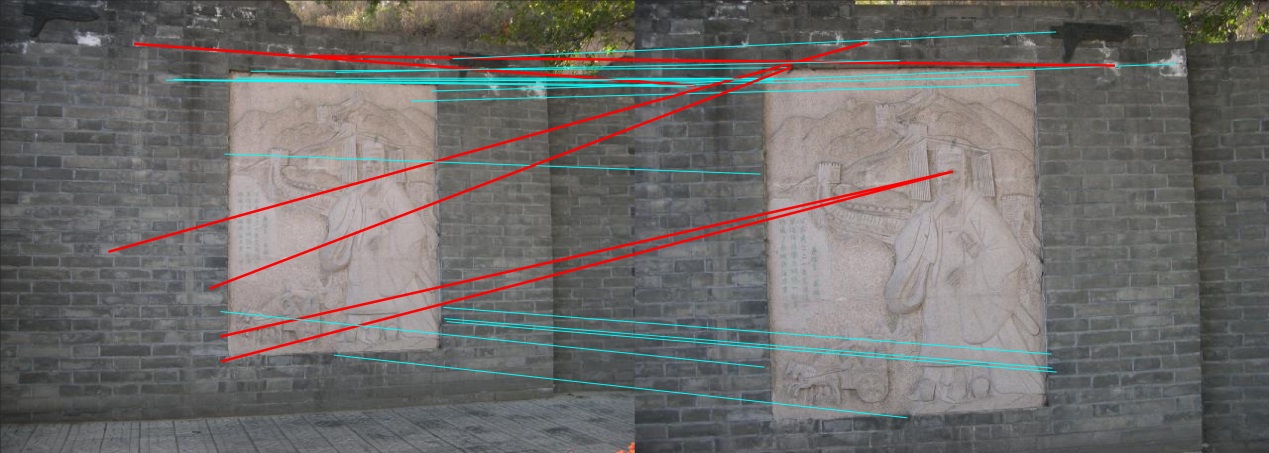


(c) FREAK


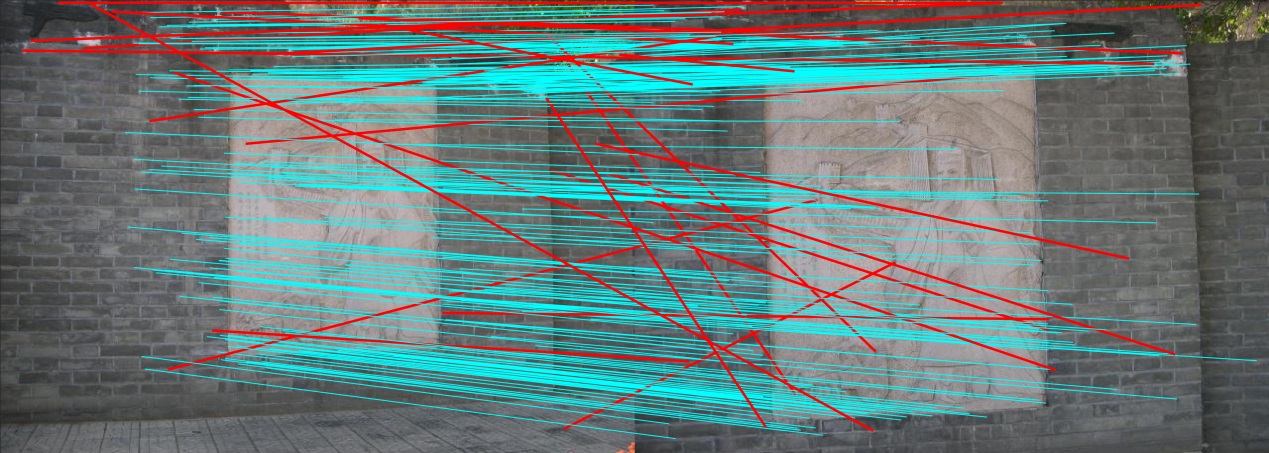


(c) SIFT


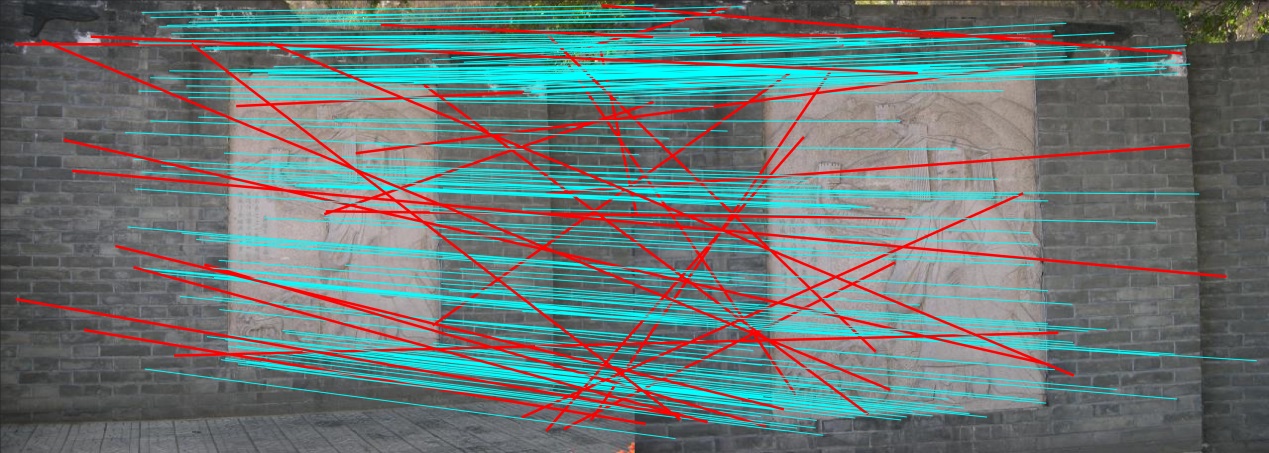


(c) Chen’s method


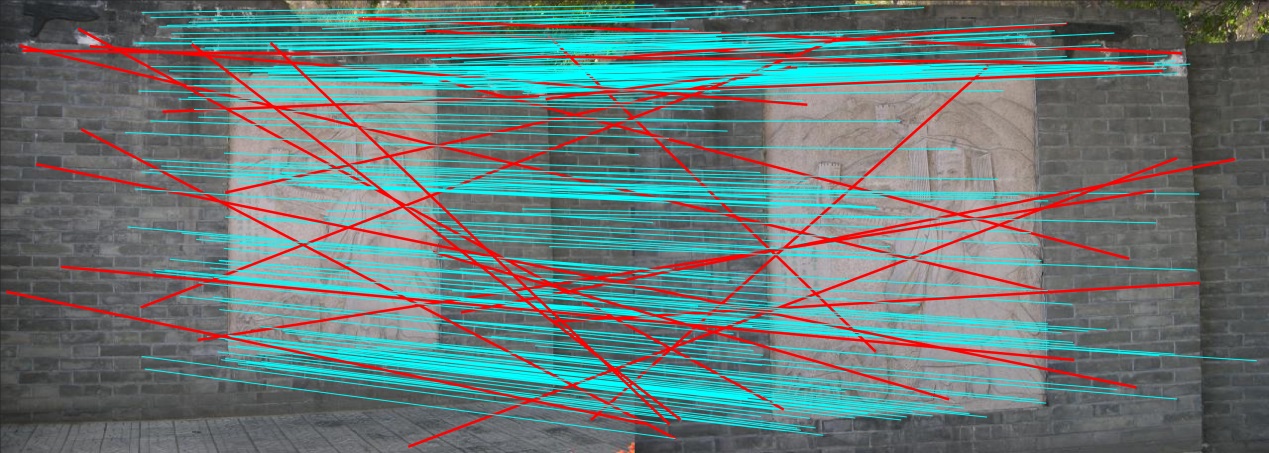


(c) Zhou’s method


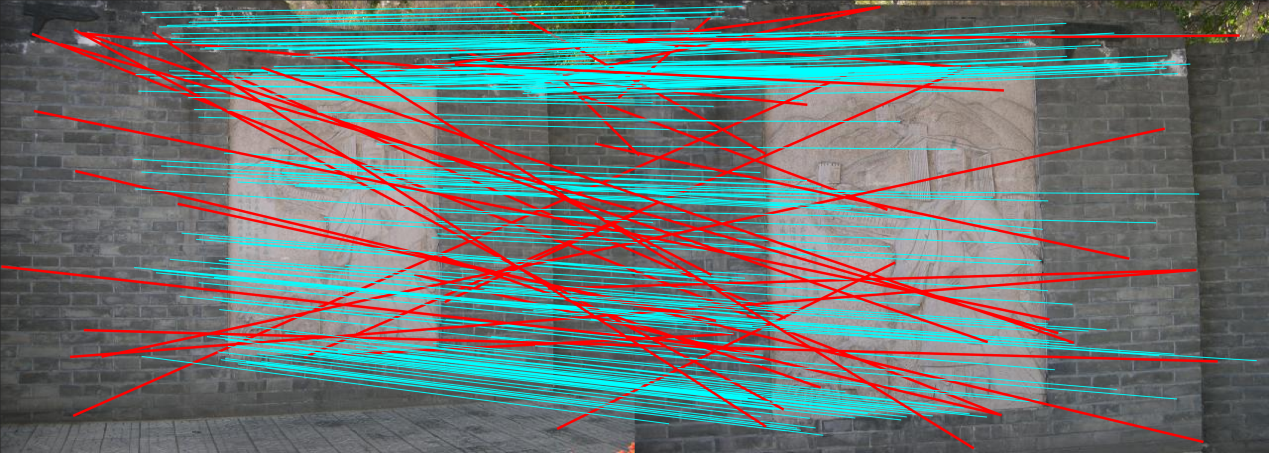


(c) MBR-SIFT’


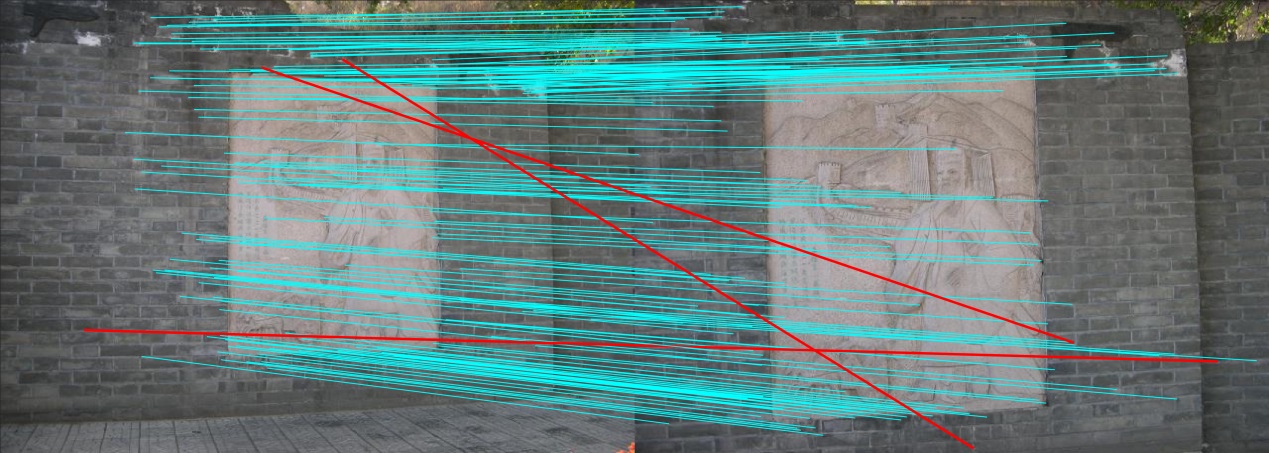


(c) MBR-SIFT


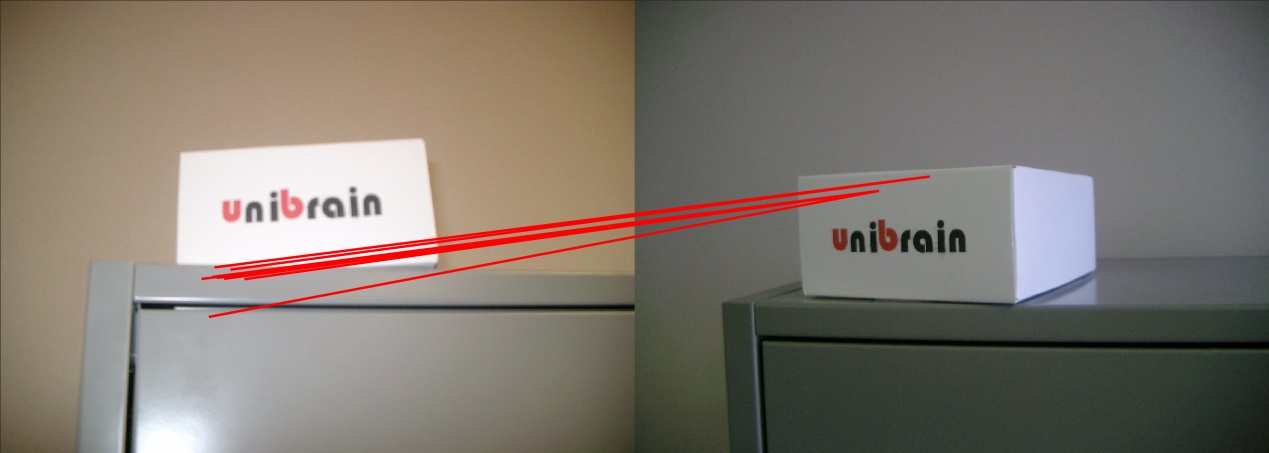


(d) CS-LBP


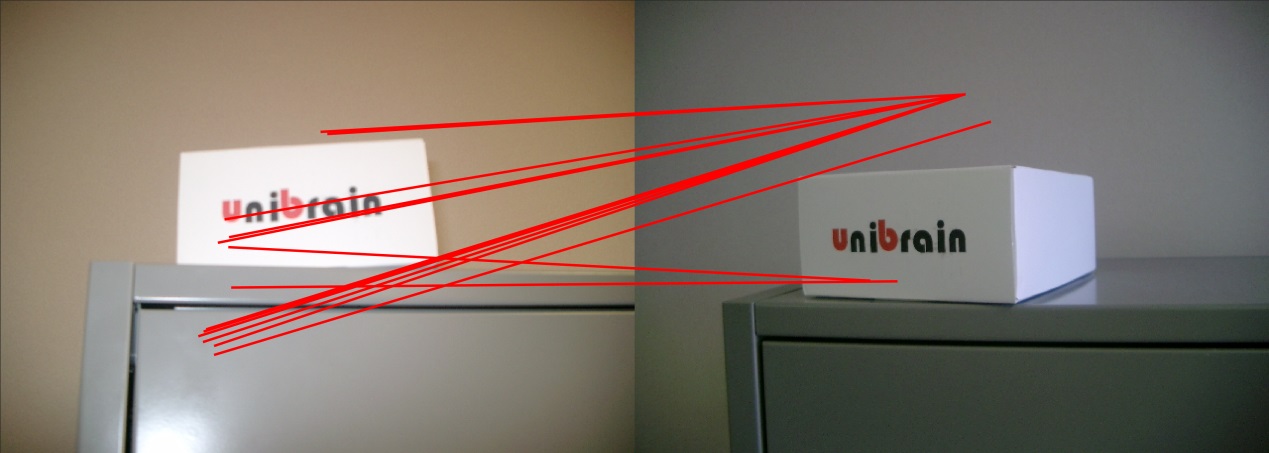


(d) BRIEF


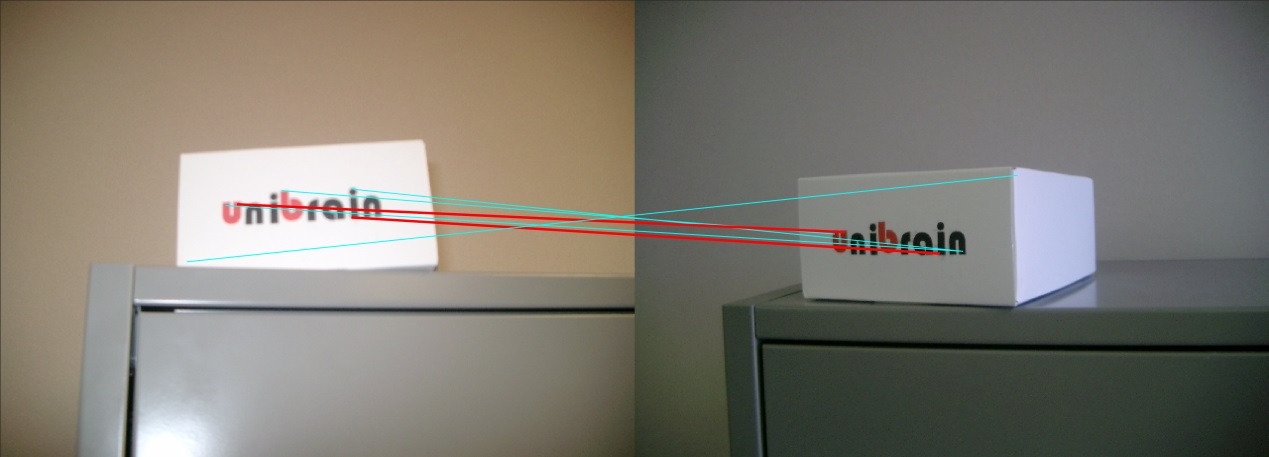


(d) BRISK


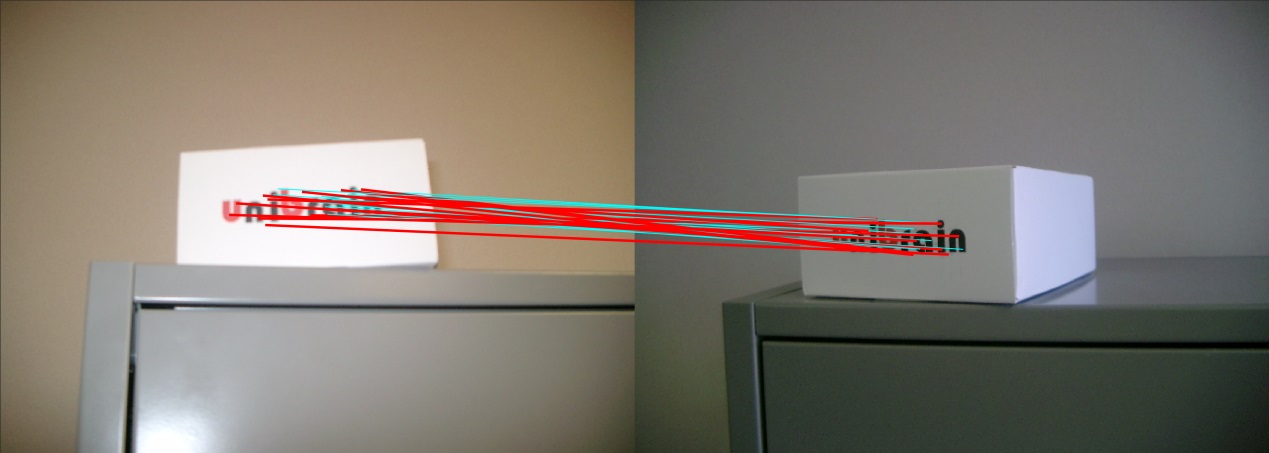


(d) FREAK


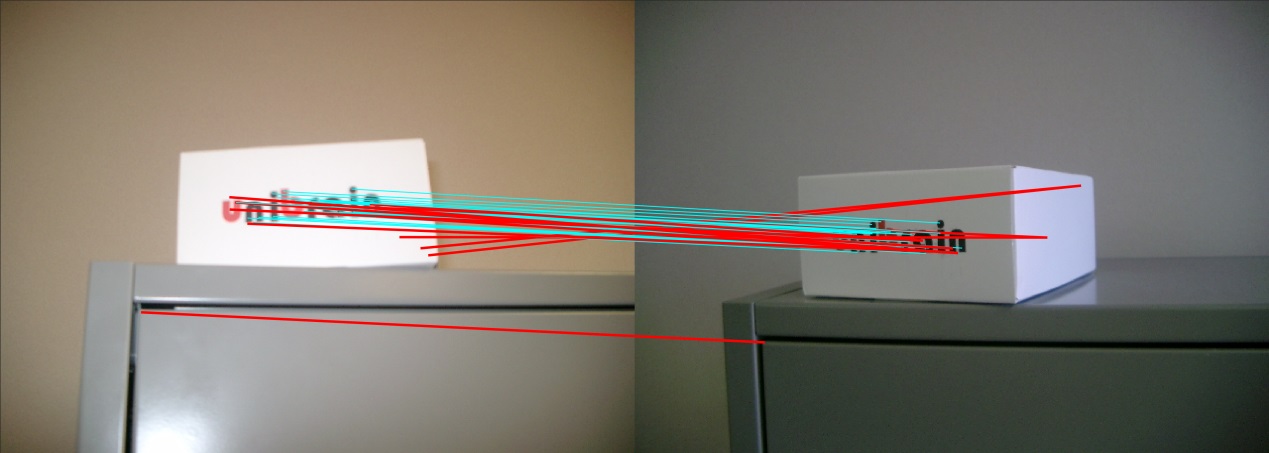


(d) SIFT


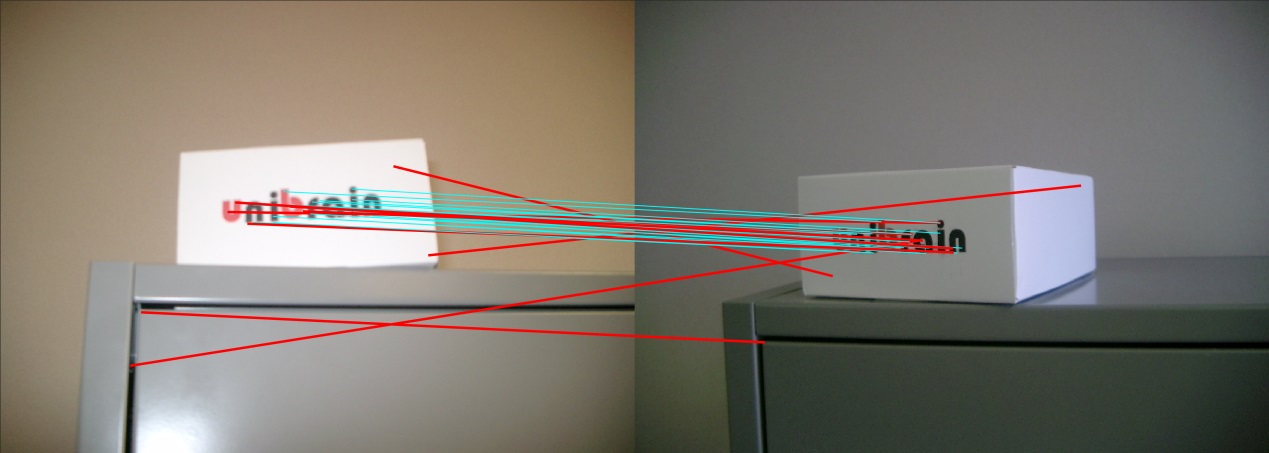


(d) Chen’s method


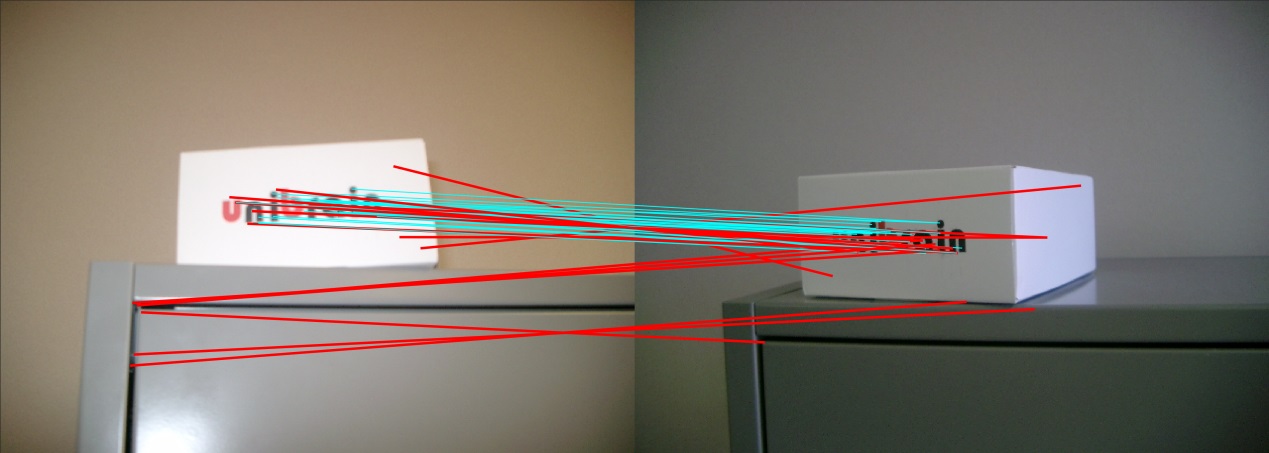


(d) Zhou’s method


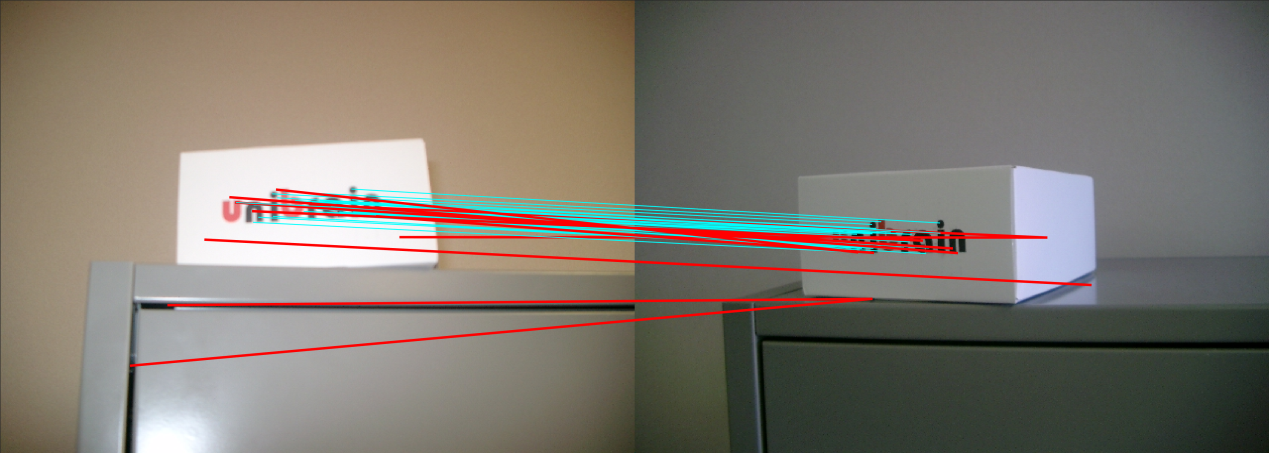


(d) MBR-SIFT’


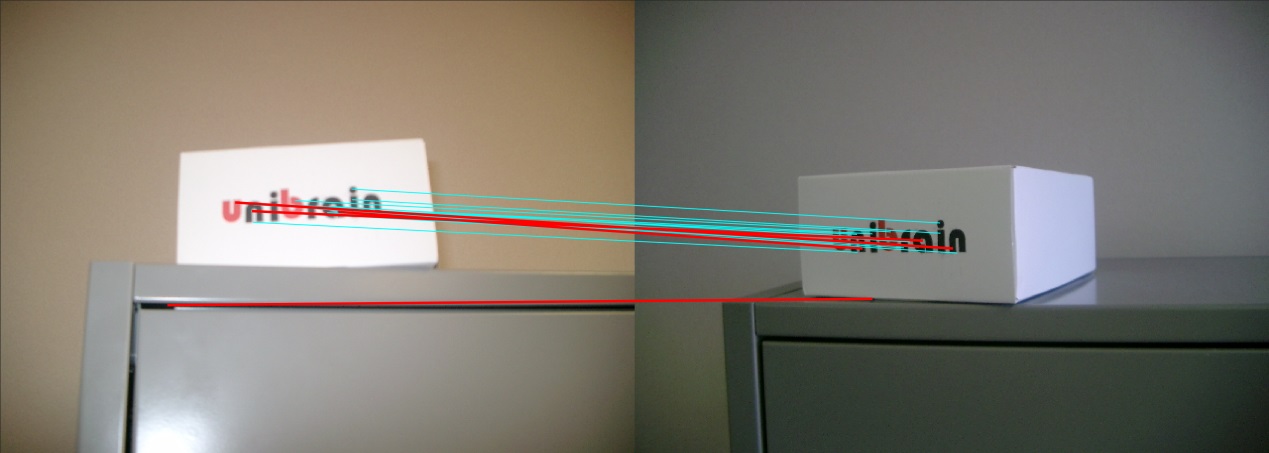


(d) MBR-SIFT


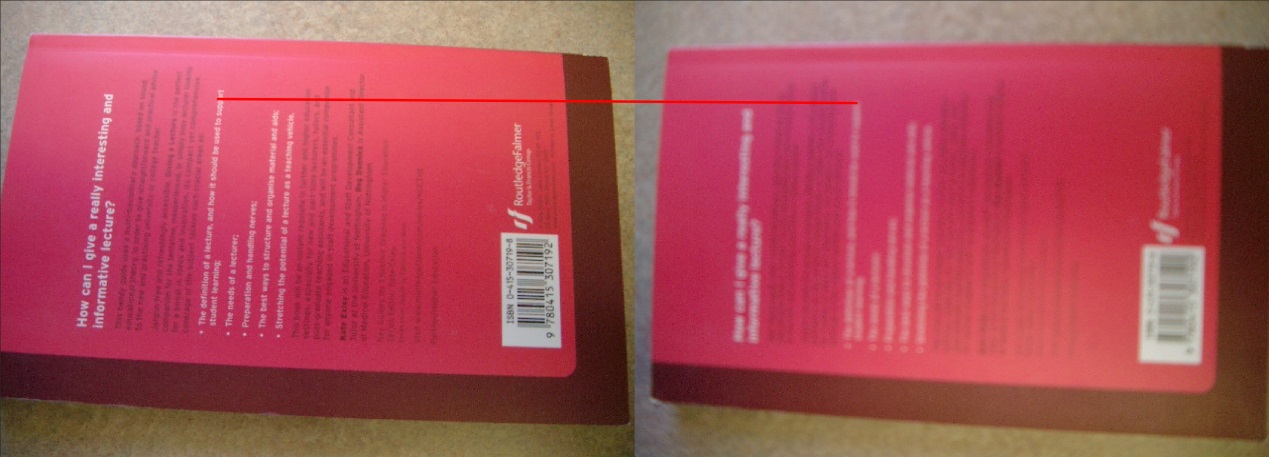


(e) CS-LBP


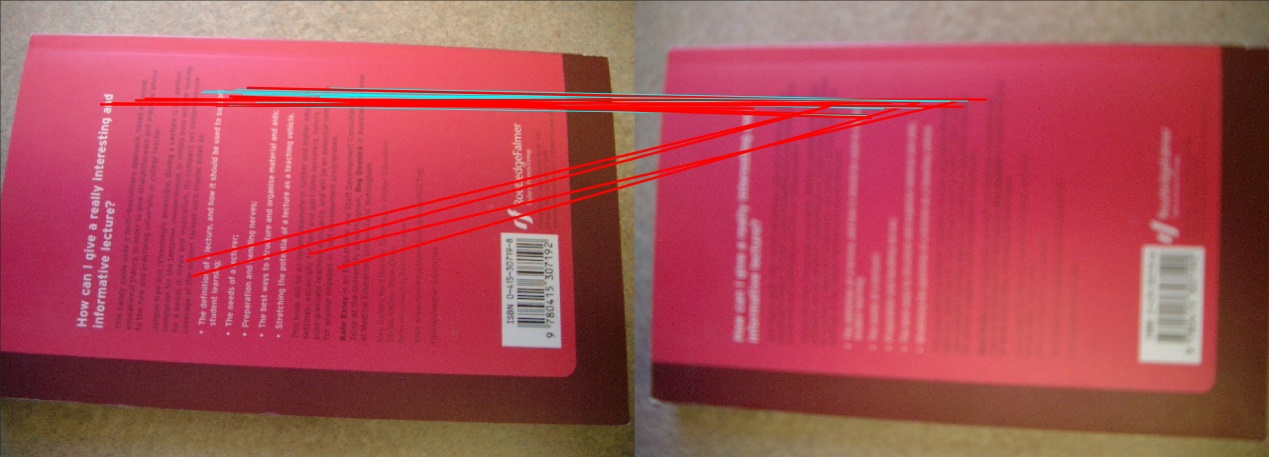


(e) BRIEF


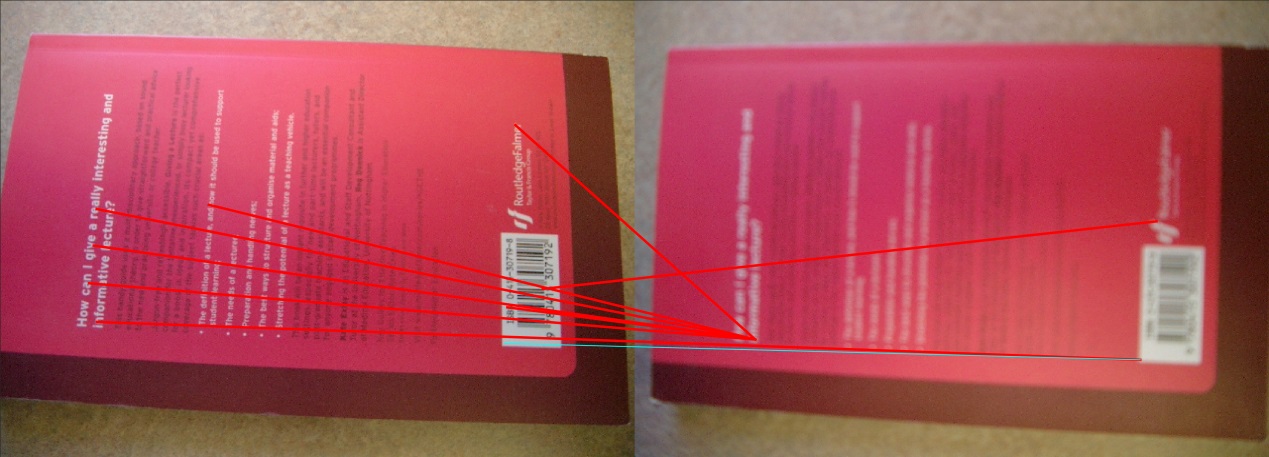


(e) BRISK


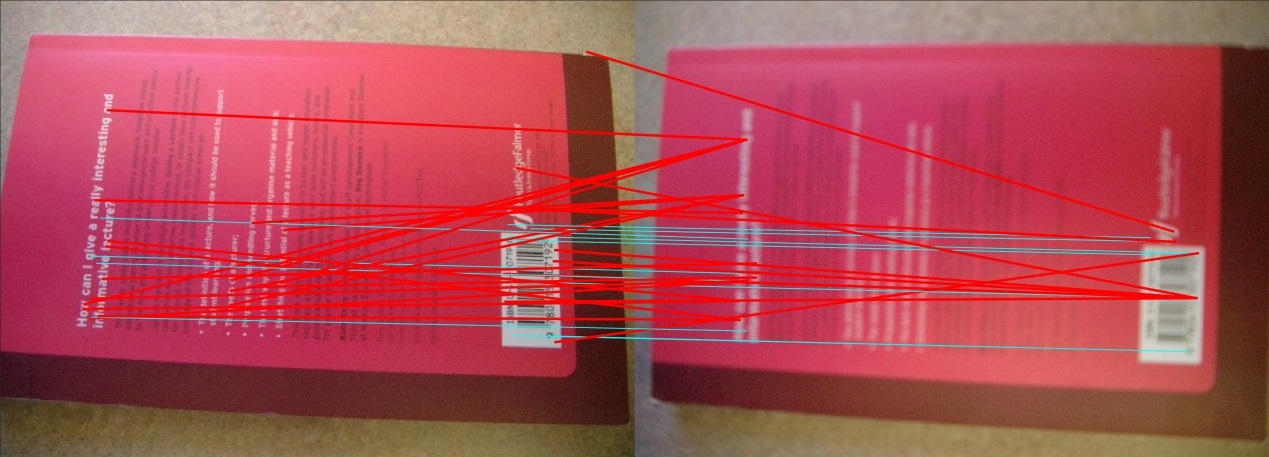


(e) FREAK


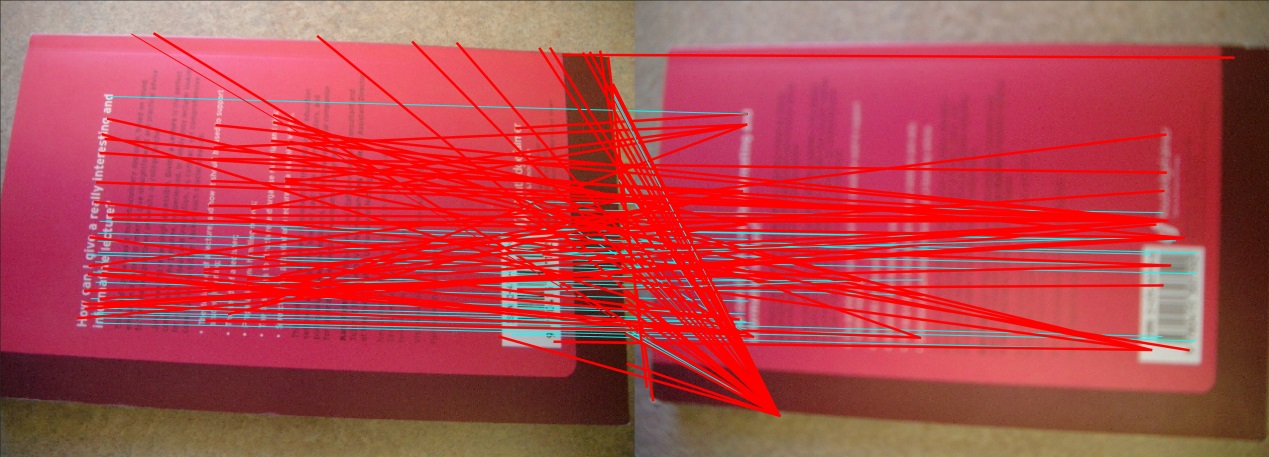


(e) SIFT


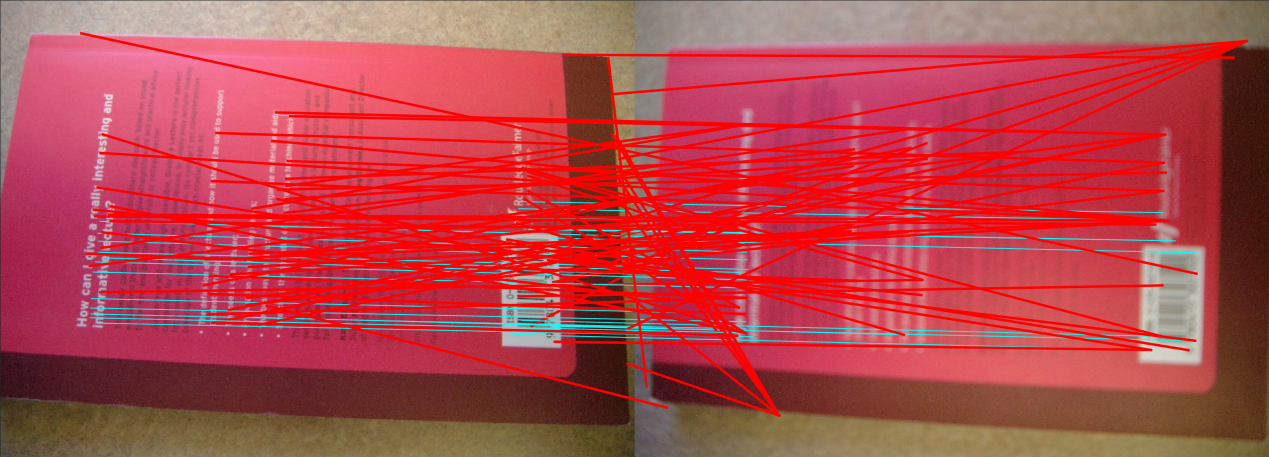


(e) Chen’s method


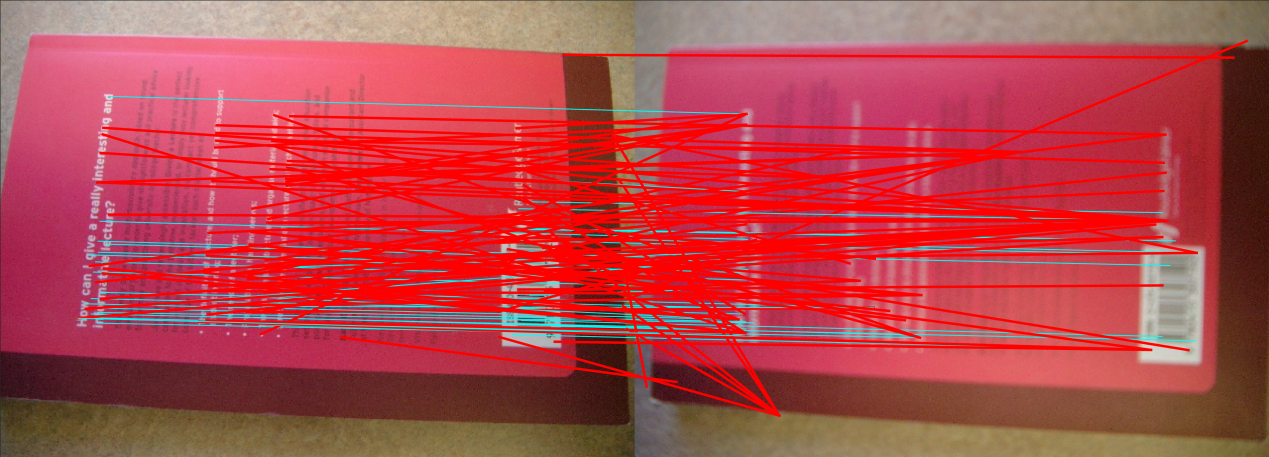


(e) Zhou’s method


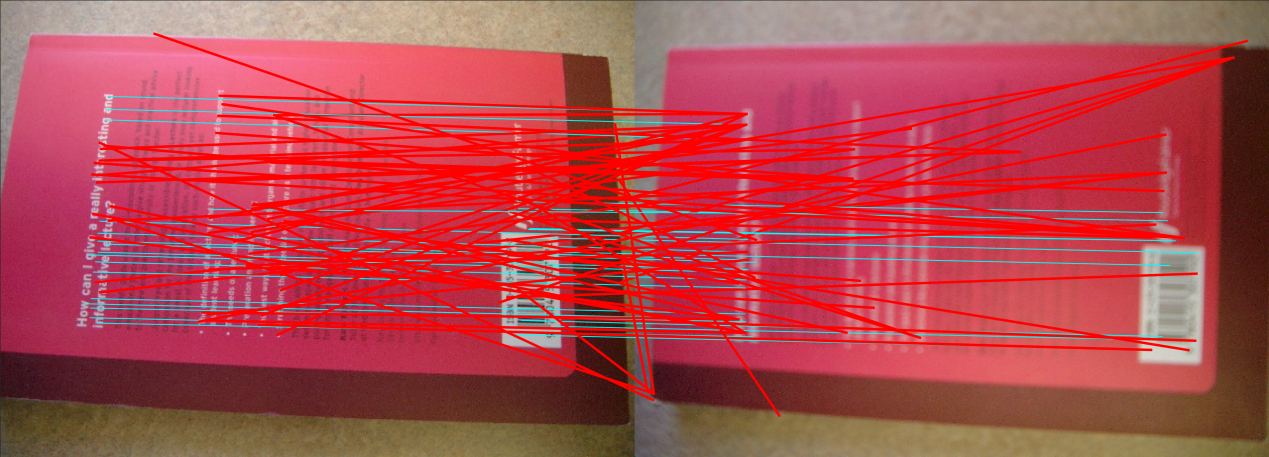


(e) MBR-SIFT’


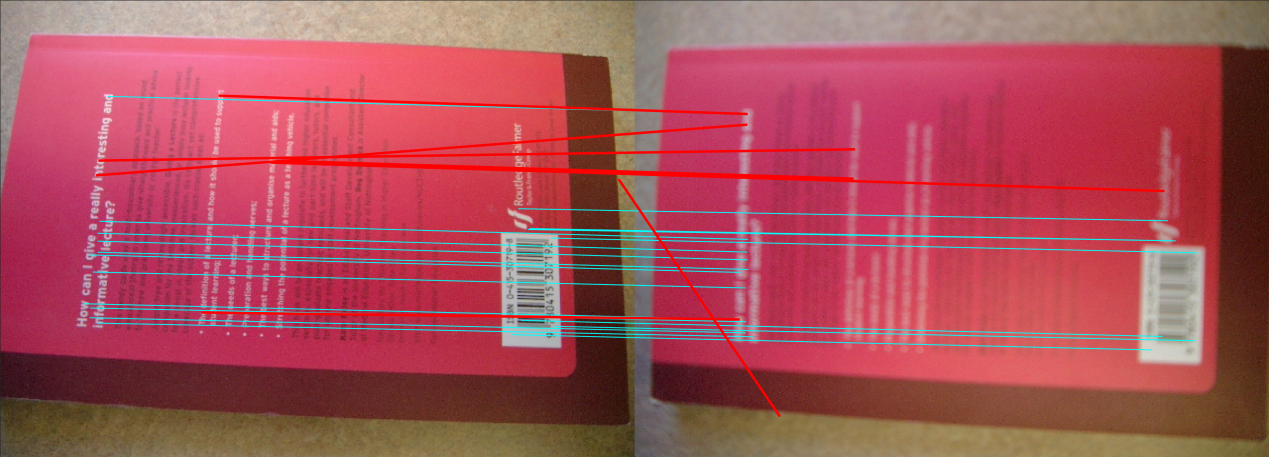


(e) MBR-SIFT

**Fig. Comparing the matching performance for CS-LBP, BRIEF, BRISK, FREAK, SIFT, Chen’s method, Zhou’s method, MBR-SIFT’ and MBR-SIFT.**
